# Structure–Property Relationships of Elastomeric Vinylogous Urethane Thermosets and Their Application as Closed-Loop Recyclable Strain Sensors

**DOI:** 10.1021/acs.macromol.4c03256

**Published:** 2025-02-05

**Authors:** Youwei Ma, Francesco Stellacci

**Affiliations:** 1 Institute of Materials, École Polytechnique Fédérale de Lausanne (EPFL), Lausanne 1015, Switzerland; 2 Institute of Bioengineering, École Polytechnique Fédérale de Lausanne (EPFL), Lausanne 1015, Switzerland

## Abstract

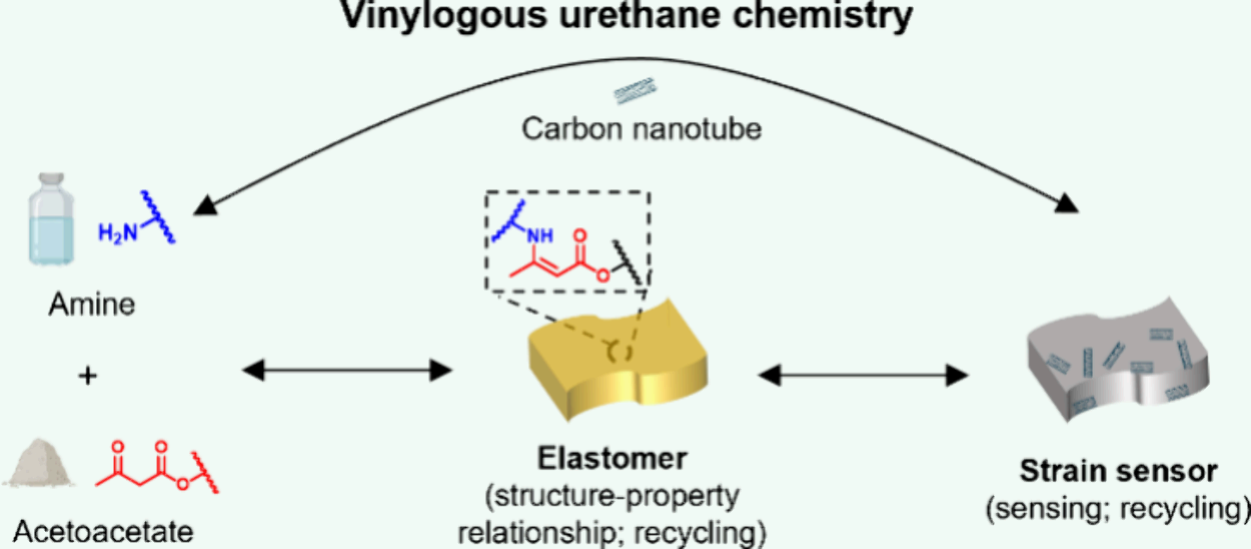

Developing closed-loop recyclable thermosets and understanding their structure–property relationships are essential steps in advancing a circular materials economy. Here, we present a vinylogous urethane (**VU**) thermoset with closed-loop recyclability, synthesized through the reaction of polytetrahydrofuran bisacetoacetate (**aPTHF**) and tris(2-aminoethyl)amine (**TREN**). These **VU** polymers exhibit high elasticity, with only a 3–9% residual strain observed after cyclic tensile testing at a maximum strain of 100%, depending on the molecular weight of **aPTHF** and network cross-link density. The two structural parameters also allow modulation of the mechanical and stress-relaxation properties of **VU** elastomers. To investigate the hydrolysis of the **VU** linkages within the hydrophobic **aPTHF** matrix, we employed a heterogeneous system using a biphasic mixture of HCl and CDCl_3_. Our findings show that the hydrophobic **VU**s remain stable in pure water but can be dissociated under acidic conditions, with the dissociation rate accelerated at higher temperatures and/or in the presence of higher HCl concentrations. These detailed investigations indicate the potential of **VU** elastomers as sustainable substrates for wearable sensors. We therefore conduct a case study of synthesizing a strain sensor through the incorporation of multiwalled carbon nanotubes (**MCN**s) into the **VU** elastomer matrix. The sensor can robustly detect various movements. Moreover, acidic treatment of both the neat polymer and the sensor composite using a HCl and diethyl ether solvent mixture allows for the excellent recovery of **aPTHF** (>90%) and **TREN** (86%), without discernible damage to the **MCN**s reclaimed from the latter.

## 1 Introduction

Thermosets, with a cross-linked architecture, are known for their excellent mechanical properties, thermal resistance, and dimensional stability, rendering them integral in diverse applications.^1,2^ However, the cross-linked structure inherently precludes their reprocessing and reuse, unlike thermoplastic polymers, possessing a linear configuration that allows thermal reprocessing.^3,4^ Consequently, post-use thermosets are frequently disposed of in landfills, incinerated, or subjected to downcycling through mechanical and thermochemical recycling processes, leading to substantial economic losses and environmental pollution.^5,6^ Therefore, developing efficient recycling methods for thermosets is of crucial importance.

Recent advancements in dynamic covalent chemistries offer a promising solution to the recycling challenges faced by thermosets.^4,7−10^ This approach involves designing thermosets with dynamic covalent linkages capable of undergoing reversible cleavage and reformation (dissociative pathway) or exchanging with one another (associative pathway). These features allow heat-assisted and/or depolymerization-induced recycling of the thermosets after their lifespan.^11−15^ The former recycling approach utilizes industrially relevant reprocessing techniques, such as thermal compression and melt extrusion, and triggers exchange reactions at high temperatures.^9,16^ The depolymerization-induced recycling consists of the initial depolymerization of thermosets into their constituent components and the subsequent repolymerization into materials of comparable properties to the pristine ones (i.e., closed-loop recycling).^17,18^ These two recycling approaches can be complementary, in which closed-loop recycling is particularly advantageous in targeting high-temperature-sensitive polymers and/or composites comprising nonflowable fillers^8,19^ as it proceeds at lower temperatures and allows the separation of composite fillers after depolymerization.

Dynamic covalent linkages that enable both reprocessing and closed-loop recycling of the thermosets have recently attracted significant attention, with the examples of cyanurate,^4^ tri/diketoenamines,^20−22^ amide-derived linkages,^11,12,19,23−25^ vinylogous urethanes (**VU**s),^8,26−33^ and dioxaborolanes,^34,35^ to name a few. Among them, the **VU** chemistry—involving the reaction of acetoacetates and amines—reported by the Du Prez group in 2015,^27,29^ is of our particular interest since it exhibits high modularity and synthetic accessibility. Ever since, it has been investigated extensively, ranging from development of novel polymer materials and alternative synthetic strategies (such as amino-yne “click” polyadditions) to applications,^31,33,36−45^ with the initial focus on recycling the **VU** polymer networks through thermal reprocessing techniques or small molecule amine-induced depolymerization.^46−48^ A recent work from Ma et al. revealed that the **VU** polymer networks comprising hydrophilic polyethylene glycol skeletons could be depolymerized/hydrolyzed in the presence of excess water, allowing for near-quantitative recovery of the starting components.^8^ In contrast, **VU** linkages in highly hydrophobic polymers exhibited significant resistance to dissociation and yet were cleaved under acidic conditions.^8^ The environmentally benign nature of (acidic) water soon encouraged several studies adopting this method to recycle **VU** polymer networks.^26,49−51^ However, the hydrolysis behavior and structure–property relationships of the hydrophobic **VU** polymers featuring the polytetrahydrofuran bisacetoacetate (**aPTHF**) skeletons remain unexplored in that work.^8^

The Du Prez and Winne laboratories have systematically investigated the structure–property relationships of various **VU** polymer networks, including those incorporated with **aPTHF** skeletons,^52^ with a particular interest in the elucidation of their viscoelastic properties (encompassing creep and stress relaxation) by adjusting the relevant structural parameters, such as the dynamics of **VU**-amine exchange,^28,29,32,37,38,53,54^ chain length^44^ and rigidity,^30,52^ and network cross-link density.^27,31,45^ The exquisite knowledge established by them points to controlling the dynamic behavior of resulting **VU** polymers on demand, sometimes enabling both fast reprocessing and creep-resistance—features often considered mutually exclusive in dynamic covalent networks.^37,38,53−56^ Of equal importance are other aspects of material’s properties, particularly the mechanical properties, when assessing the overall performance of a material. We anticipate that a detailed investigation into these aspects will further catalyze the application of **VU** polymers in more specific (even value-added) fields.

**Scheme 1:**
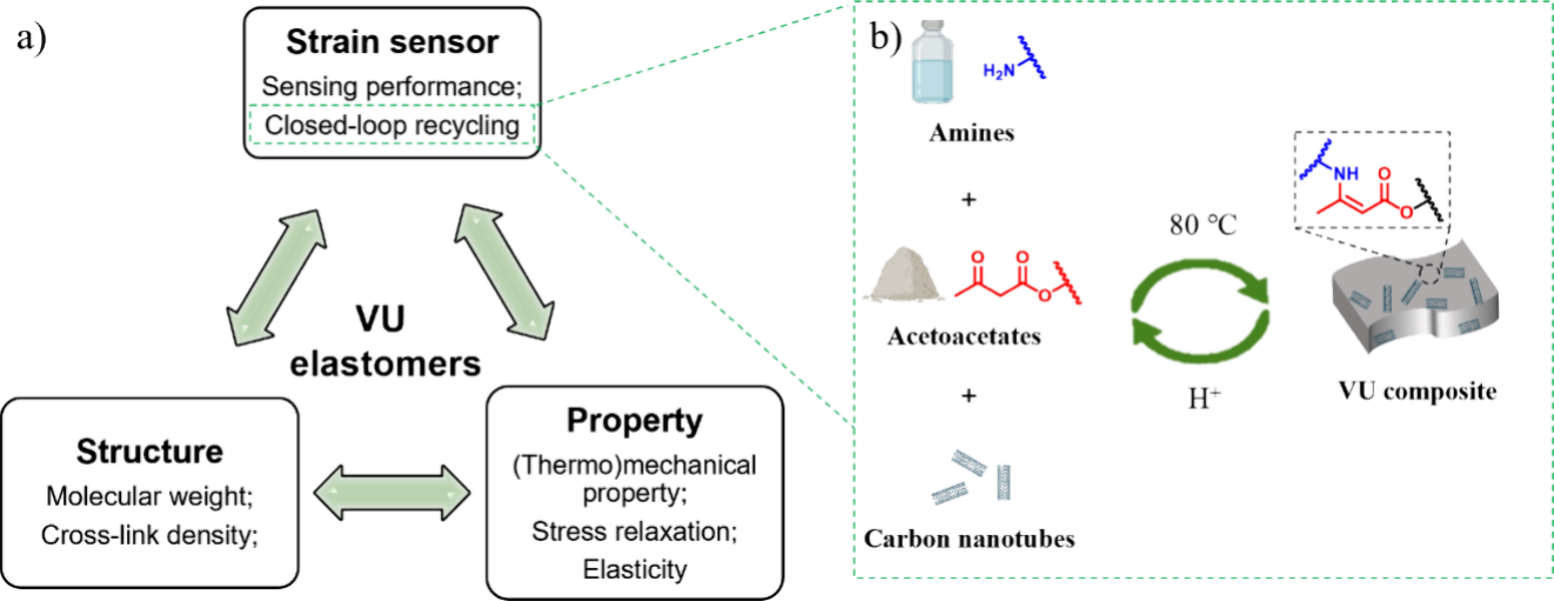
(a) General Overview of the Investigations Conducted in This Study; (b) Illustration of the Synthesis of Vinylogous Urethane (**VU**)/Carbon Nanotube Composite from the Mixture of Amines, Acetoacetates, and Carbon Nanotubes in Which the Reaction of the Former Two Components Leads to the Formation of **VU** Linkages Followed by the Depolymerization of the **VU** Composite into Its Starting Materials upon Acidic Treatment

With this in mind, we sought to elucidate the structure–property relationships of **VU** polymer networks more comprehensively. The **aPTHF**-based **VU** polymers, as reported in the works by Du Prez, Winne, Guerre, and co-workers^52^ and also Ma et al.^8^ were first synthesized through the reaction of **aPTHF** and excess tris(2-aminoethyl)amine (**TREN**). Upon evaluation of their properties (including thermal, mechanical, and thermomechanical behaviors, stress relaxation, and elasticity) by adjusting the structural parameters such as the molecular weight of **aPTHF** and network cross-link density (Scheme 1a), their relationships were established. One finding shows that the **VU** polymers are a type of elastomer and exhibit excellent elasticity. This feature motivates us to explore their potential in strain sensor applications, specifically synthesized by incorporating multiwalled carbon nanotubes (**MCN**s) into the reaction mixture of **aPTHF** and **TREN** (Scheme 1b). Such motivation also arises from the significant socioeconomic impact of strain sensors, a fast-growing field currently facing the challenge of effective management on their postconsumer polymeric substrates.^57−60^

The hydrolysis of dynamic covalent linkages, **VU** included, incorporated within hydrophobic substrates has been demonstrated in some studies using solvent mixtures;^8,26,49,61^ however, its kinetic details, to our knowledge, have yet to be tapped. This gap is primarily due to the limited solubility of hydrophobic substrates, which impedes effective interactions between water and hydrolyzable motifs. From a chemical engineering perspective, investigating the kinetics of this process is essential for optimizing energy and resource efficiency by reducing temperature and minimizing the quantities and concentrations of hydrolyzing agents, which is particularly relevant in large-scale manufacturing. To this end, we carried out a small-molecule model study using a biphasic mixture of HCl and CDCl_3_ to investigate the hydrolysis of a hydrophobic **VU** compound, specifically bis(butyl acetoacetate)-terminated polytetrahydrofuran (**aPTHF-Btl**). Exerting control over parameters including water content, acid concentration, and temperature allowed us to determine their impacts on the kinetic and thermodynamic aspects of the **VU**’s dissociation. The insight thus gained then translates to depolymerizing the **aPTHF**-based **VU** elastomers and their strain sensor composite, allowing for the excellent recovery of **aPTHF** and **TREN**, and also **MCN**s in the case of the composite (Scheme 1b).

## 2 Experimental Section

### 2.1 Materials

Polytetrahydrofuran (**PTHF**, *M*_n_ = 1000, 2000, 2900 g mol^–1^), tris(2-aminoethyl)amine (96%, **TREN**), *tert*-butyl acetoacetate (98%, **tBA**), chloroform-*d* (99.8%, CDCl_3_), diethyl ether, multiwalled carbon nanotubes (98%, **MCNs**), *n*-butylamine (99.5%, **Btl**), and strongly acidic ion-exchange resin (50 to 100 mesh, DOWEX 50WX8–10) were purchased from Sigma-Aldrich.

### 2.2 Synthesis of Polytetrahydrofuran Bisacetoacetate (aPTHF_*x*_)

Commercial **PTHF**_*x*_ (10 g) was weighed and added to a 50 mL single-necked flask together with a stirring bar. The flask was equipped with a condenser and a Dean–Stark apparatus to remove the *tert*-butanol generated during the reaction. Then, **tBA** (10 equiv) was added in one portion with a pipet, and the resulting mixture was heated at 155 °C for 3 h. The unreacted **tBA** was removed by vacuum distillation at 100 °C for 12 h to afford **aPTHF**_*x*_.

### 2.3 Synthesis of bis(Butyl acetoacetate)-Terminated Polytetrahydrofuran (aPTHF_2k_-Btl)

**Btl** (0.73 g, 10 mmol) was combined with **aPTHF**_**2k**_ (2.2 g, 1 mmol) in 10 mL of THF. The reaction mixture was heated to 60 °C for 24 h while stirring. The excess of **Btl** and THF was removed by rotary evaporation followed by subsequent vacuum drying at 70 °C for 12 h. **aPTHF**_**2k**_**-Btl** was obtained as a yellowish liquid (2.2 g; yield 95%).

### 2.4 Synthesis of Vinylogous Urethane Polymer Networks and the Strain Sensor

In a typical procedure to synthesize the **VU** polymer, **aPTHF**_*x*_ (1.2 g for **aPTHF**_**1k**_, 2.2 g for **aPTHF**_**2k**_, and 3.1 g for **aPTHF**_**2.9k**_) was dissolved in 10 mL of ethanol in a 20 mL vial. Then, **TREN** (0.127 g, 0.87 mmol) was added to the mixture. The resulting solution was stirred for 10 min at room temperature and then poured into a Teflon mold. The mixture was heated at 60 °C overnight in a drying oven (no vacuum), and then it was dried by applying a vacuum at 80 °C for 12 h. A yellowish solid was obtained, which was labeled as **aPTHF**_*x*_**-yTREN**. **VU** polymers from the reaction of **aPTHF**_**2k**_ and different amounts of excess amine (10, 30, and 50 mol %) were prepared by adjusting the molar ratio of **TREN** added.

The synthesis of strain sensor (labeled as **VU/5%MCNs**) follows a similar procedure, involving the addition of **MCNs** (0.116 g) into the reaction mixture of **aPTHF**_**2k**_ (2.2 g, 1 mmol) and **TREN** (0.127 g, 0.87 mmol).

### 2.5 Depolymerization of aPTHF_*x*_-1.3TREN and the Strain Sensor and Subsequent Recovery of Starting Materials

0.5 g of **aPTHF**_*x*_**-1.3TREN** shreds (*x* = 1k, 2k, 2.9k) was added to 15 mL of 1 M HCl in a small vial while stirring at a rate of 400 rpm. After 18 h, 15 mL of diethyl ether was added to the depolymerized mixture, and the resulting two-phase mixture solution was stirred at 400 rpm for another 24 h. It resulted in a clear two-phase mixture solution. To recover **aPTHF**_*x*_, the upper phase ether solution was removed by using a separation funnel. The separated solution was washed with pure water three times and then dried with magnesium sulfate (MgSO_4_). MgSO_4_ was filtered out, and the diethyl ether was removed by rotary evaporation. After being vacuum-dried at 80 °C for 24 h, yellowish liquid (**aPTHF**_**1k**_) or solid (**aPTHF**_**2k**_ and **aPTHF**_**2.9k**_) materials were obtained.

To recover **TREN**, for the sake of operation, the bottom 1 M HCl solution was collected and combined together from these three vials. The obtained solution was washed with diethyl ether gently three times. Next, ca. 45 mL of 1 M NaOH was added to the acid solution until it became slightly alkaline, determined by a pH paper. The resulting solution was dried by rotary evaporation before 10 mL of CHCl_3_ was added to extract the organic compound from the mixture. Finally, the obtained CHCl_3_ solution was filtered using a syringe filter, and the CHCl_3_ was removed by rotary evaporation, leading to the formation of a yellowish liquid crude. **TREN** was recovered by vacuum drying the crude at 60 °C for 24 h.

The depolymerization of the **VU/5%MCNs** composite was carried out by treating 0.5 g of **VU/5%MCNs** with a two-phase mixture solution comprising 10 mL of 1 M HCl and 10 mL of diethyl ether. The mixture was kept at room temperature for 24 h while being stirred at a rate of 400 rpm. After that, the **MCNs** could be recovered by vacuum filtration. The upper diethyl ether phase of the filtrate was then taken out using the separation funnel to recover **aPTHF**_**2k**_.

Detailed experimental procedures are also provided in the Supporting Information.

## 3 Results and Discussion

### 3.1 Model Studies on Dissociation Behavior of Hydrophobic Vinylogous Urethane

Polytetrahydrofuran (**PTHF**_*x*_) with an *M*_n_ of 1000 (**PTHF**_**1k**_), 2000 (**PTHF**_**2k**_), or 2900 g mol^–1^ (**PTHF**_**2.9k**_) was selected as the hydrophobic matrix and first converted into polytetrahydrofuran bisacetoacetate (**aPTHF**_*x*_) by a transesterification reaction between **PTHF**_*x*_ and 10 equiv of *tert*-butyl acetoacetate (**tBA**). The reactions were carried out at 155 °C for 3 h with the removal of unreacted **tBA** by vacuum distillation at 100 °C for 12 h for reuse. The reactions afforded **aPTHF**_*x*_ in yields of 91–98% and with the extent of conversion ranging from 97% to 100%, as determined by the weight and integration of the ^1^H NMR signals of purified **aPTHF**_*x*_, respectively (Figures S2–S4; calculations on page S4). Thereinto, **aPTHF**_**2k**_ was used to synthesize bis(butyl acetoacetate)-terminated polytetrahydrofuran (**aPTHF**_**2k**_**-Btl**; ^1^H and ^13^C NMR spectra in Figures S6 and S7) by a condensation reaction with butylamine (**Btl**) upon thermal treatment at 60 °C for 24 h, which served as the model compound.

**Figure 1:**
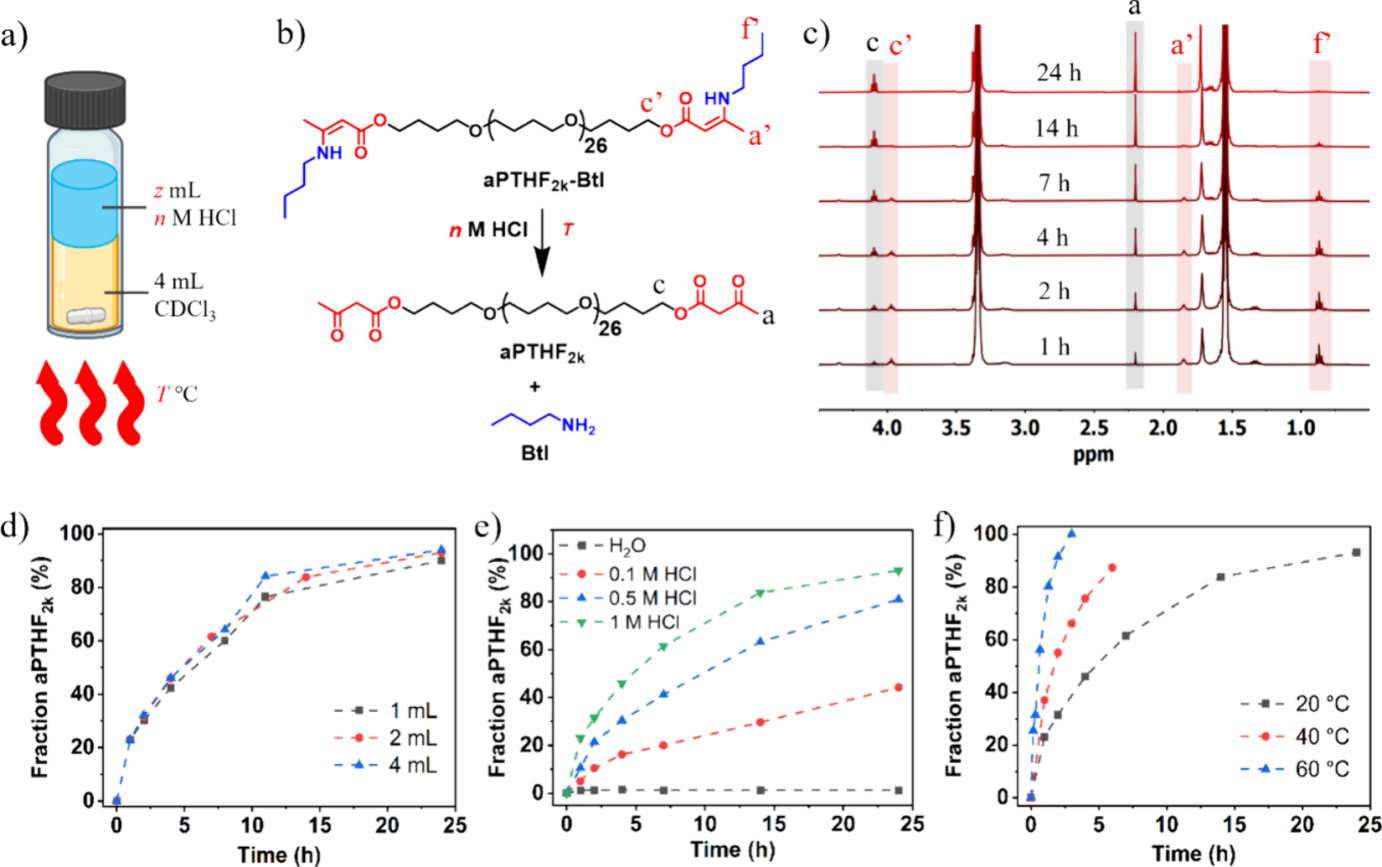
(a) Schematic illustration showing a biphasic mixture solution comprising *z* mL of *n* M HCl (upper phase) and 4 mL of CDCl_3_ (bottom phase) upon thermal treatment at temperature *T*, used for the investigation of the dissociation of **aPTHF**_**2k**_**-Btl**. (b) The dissociation of **aPTHF**_**2k**_**-Btl** into **aPTHF**_**2k**_ and **Btl**, with the assignment of the key protons shown in the ^1^H NMR spectra. (c) Partial ^1^H NMR spectra recorded during the dissociation of **aPTHF**_**2k**_**-Btl** (200 mg) in the presence of the mixture solution of 1 M HCl (2 mL) and CDCl_3_ (4 mL) at 20 °C. Extent of conversion of **aPTHF**_**2k**_**-Btl** into **aPTHF**_**2k**_ and **Btl**, measured as a function of time with HCl solution used in (d) different amounts (1, 2, 4 mL) and (e) different concentrations (0, 0.1, 0.5, 1M), or at (f) different temperatures (20, 40, 60 °C).

A technical difficulty of investigating the dissociation of hydrophobic **aPTHF**_**2k**_**-Btl** (flanked by two **VU** motifs) in acidic aqueous solutions arises from its poor solubility in such environments. To overcome this limitation, we devised a heterogeneous system to carry out the model reactions. As illustrated in Figure 1a, the system employs a biphasic mixture with HCl as the upper phase and CDCl_3_ as the lower phase. The **aPTHF**_**2k**_**-Btl** dissolves in the CDCl_3_ phase, and while stirring, HCl can catalyze the hydrolysis of **VU** linkages at the interface between the two phases (Figure 1b). Exerting control over parameters including reaction temperature and the amount and concentration of HCl allowed us to evaluate their individual impact on the dissociation.

Unlike the homogeneous systems adopted in most model studies,^7,27,35,62,63^ where both the model compounds and dissociation agents dissolve in the same solvent for full molecular interaction, our setup relies on reactions occurring at the interface between two immiscible solvents (i.e., CDCl_3_ and HCl). First and foremost, we assessed the feasibility of the dissociation reaction by treating **aPTHF**_**2k**_**-Btl** (200 mg) with a 1 M HCl/CDCl_3_ mixture solution (6 mL; 1/2, v/v) at 20 °C. At different time intervals, 0.5 mL aliquots of the CDCl_3_ solution were withdrawn with a syringe and subjected to ^1^H NMR spectroscopy. As shown in Figure 1c, the intensities of signals **a’**, **b’**, and **f’** in the ^1^H NMR spectra of **aPTHF**_**2k**_**-Btl** decreased (pink areas), while the signals **a** and **c** ascribed to the acetoacetate moieties in **aPTHF**_**2k**_ intensified (gray areas), demonstrating the successful acid-catalyzed hydrolysis of the **VU** linkages.

By changing the amount of 1 M HCl (1, 2, or 4 mL) in its mixture with 4 mL of CDCl_3_ and keeping other parameters constant, we then tested if this alteration could influence the dissociation behavior of **aPTHF**_**2k**_**-Btl**. The progress in the conversion of **aPTHF**_**2k**_**-Btl** into **aPTHF**_**2k**_ and **Btl** was monitored by tracking the evolution of the ^1^H NMR signals over time (Figure S8). Integration of these signals allowed us to quantify the extent of conversion (Figure 1d). In all cases, the dissociation reactions progressed steadily, further confirming the viability of our heterogeneous system in investigating the acid-catalyzed hydrolysis of hydrophobic **VU**. Notably, increasing the amount of 1 M HCl from 1 to 4 mL did not result in significant changes in reaction rate or final conversion extent (Figure 1d), suggesting that, in the presence of excess HCl, its specific volume does not further influence the dissociation kinetics of **aPTHF**_**2k**_**-Btl**.

Since the hydrolysis proceeds at the interface of the two-phase solution, the interfacial area should play a role in dictating the reaction kinetics. Such an impact was explored by carrying out the hydrolysis reaction at different stirring rates of 100, 400, or 800 rpm (Figure S9). ^1^H NMR analysis on the reaction aliquots shows that regardless of the stirring rate, the signals attributed to **aPTHF**_**2k**_**-Btl** decrease over time while the characteristic signals of **aPTHF**_**2k**_ increase (Figure S9c–e). Integration of these signals further shows that increasing the stirring rate results in an increased hydrolysis rate. For example, the degree of hydrolysis of **aPTHF**_**2k**_**-Btl** at the stirring rate of 100 rpm was 36% after 4 h and significantly increased to 46% at 400 rpm and then to 50% at 800 rpm (Figure S9f). Evidently, increasing the stirring rate from 400 to 800 rpm contributes less to the hydrolysis compared to the increase from 100 to 400 rpm (4% vs 10%), while unfortunately associating with high energy cost. Thus, a stirring rate of 400 rpm was employed in the subsequent investigations.

Next, we investigated the influence of the HCl concentration on the hydrolysis of **aPTHF**_**2k**_**-Btl** by mixing **aPTHF**_**2k**_**-Btl** (200 mg) and a HCl/CDCl_3_ mixture solution (6 mL; 1/2, v/v) at 20 °C, with the HCl used in a concentration of 0.1, 0.5, or 1 M. For comparison, a control experiment was conducted using a H_2_O/CDCl_3_ mixture (6 mL; 1/2, v/v) to hydrolyze 200 mg of **aPTHF**_**2k**_**-Btl**. The conversion of **aPTHF**_**2k**_**-Btl** into **aPTHF**_**2k**_ and **Btl** was monitored by analyzing the ^1^H NMR signals of 0.5 mL aliquots of a CDCl_3_ solution at different reaction times (Figure S10). As shown in Figure 1e, as HCl concentration increased from 0.1 to 1 M, the extent of conversion for **aPTHF**_**2k**_**-Btl** increased by over 2-fold after 24 h, from 44% to 93%. In stark contrast, the hydrolysis of **aPTHF**_**2k**_**-Btl** by pure water was negligible, inferred by less than 1% **aPTHF**_**2k**_ detected in the ^1^H NMR spectrum of the CDCl_3_ aliquot after a 24 h reaction time. The results suggest that the HCl concentration is vital in catalyzing the hydrolysis of hydrophobic **VU**, with higher HCl concentrations accelerating the reaction, while **VU** linkages remain highly stable in pure water.

The influence of temperature on the acid-catalyzed hydrolysis of **aPTHF**_**2k**_**-Btl** was then studied by immersing 200 mg of **aPTHF**_**2k**_**-Btl** in a 1 M HCl/CDCl_3_ mixture solution (6 mL; 1/2, v/v) at different temperatures (20, 40, and 60 °C). The progress of the reactions was monitored through the observation of the ^1^H NMR signals of the CDCl_3_ solution over time (Figure S11), which subsequently allowed us to determine the extent of conversion for **aPTHF**_**2k**_**-Btl** by integrating these signals (Figure 1f). At 20 °C, the extent of conversion of **aPTHF**_**2k**_**-Btl** was 31%, increased to 55% at 40 °C, and then reached a value of 91% at 60 °C after 2 h. Another 1 h of reaction at 60 °C allowed the reaction to be almost complete. The dissociation reactions of **aPTHF**_**2k**_**-Btl** proceeded in the presence of excess 1 M HCl, thus falling under pseudo-first-order conditions. Establishing the relationship between the dissociation of **aPTHF**_**2k**_**-Btl** and time allowed us to determine the kinetic rate constants (*k*) of the reaction at different temperatures *T* (Figure S12b; Table S1). Upon increasing the reaction temperature from 20 to 60 °C, the rate constant increased from 2.95^–5^ to 3.36^–4^ s^–1^, a growth of over a magnitude, reflecting that the reaction temperature has a pronounced impact on the HCl-catalyzed hydrolysis of **aPTHF**_**2k**_**-Btl**. This temperature dependence was further analyzed by plotting ln(*k*) against 1/*T*, which allowed us to calculate the activation energy (*E*_a_) of the reaction as 57 kJ mol^–1^ (Figure S12c; Table S1). Altogether, these findings demonstrate that our heterogeneous system is effective for investigating the mechanistic details of hydrophobic yet hydrolyzable chemical motifs, which we envision will play an important role in developing and understanding chemically robust and yet recyclable polymers.

### 3.2 Synthesis, Characterization, and Mechanical Properties of Hydrophobic VU Polymer Networks

**Figure 2:**
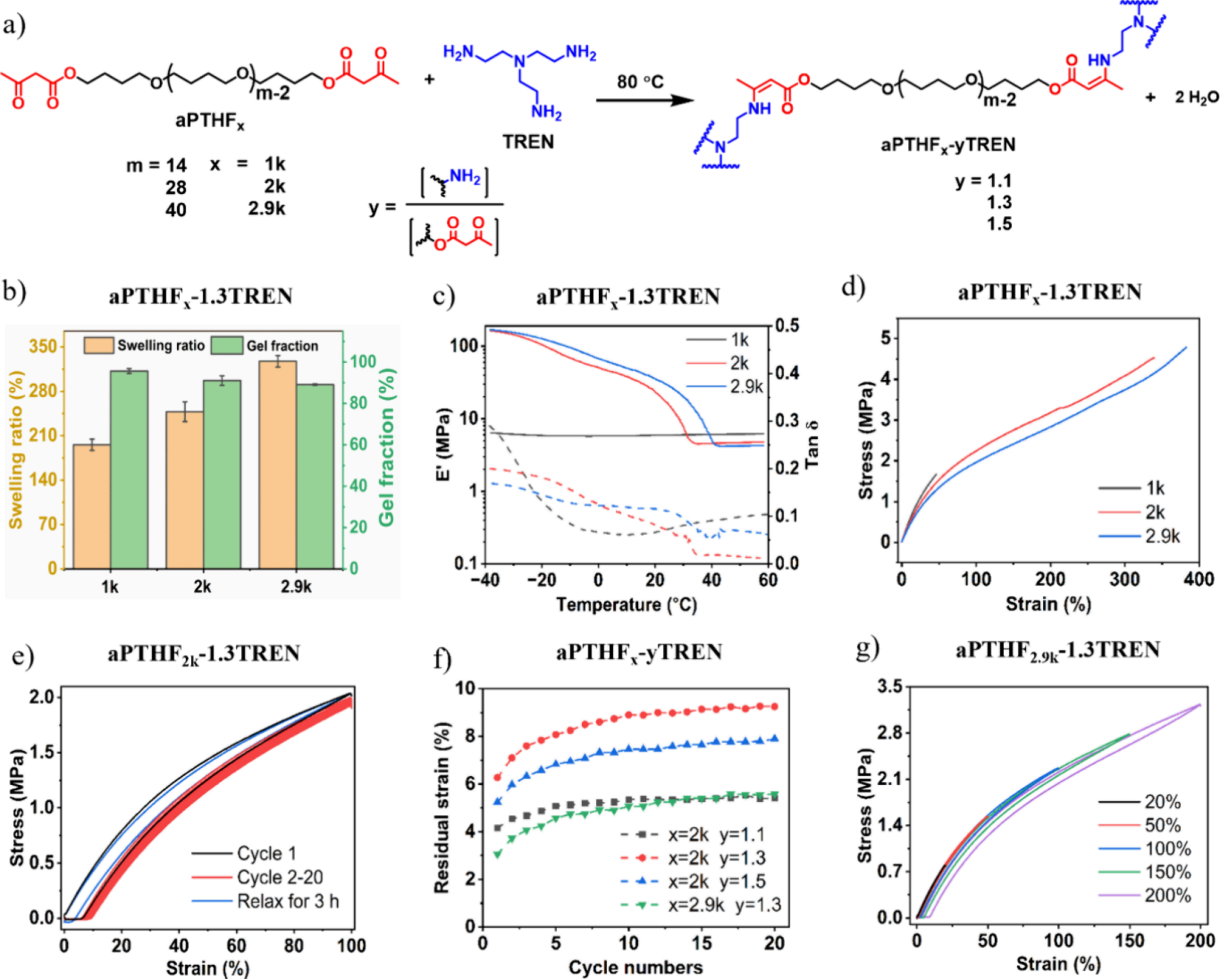
(a) Synthesis of **aPTHF**_*x*_**-yTREN** by the reaction of **aPTHF**_*x*_ and **TREN**. Parameters *x* and **y** indicate the number-average molecular weight (*M*_n_) of **aPTHF**_*x*_ and the molar ratio of amine to acetoacetate groups, respectively. (b) Swelling ratio and gel fraction, (c) DMA traces, and (d) stress–strain curves of the **aPTHF**_*x*_**-1.3TREN** series (*x* = 1k, 2k, and 2.9k). (e) Cyclic tensile curves of the 1st (black) and 2nd to 20th (red) cycles of **aPTHF**_**2k**_**-1.3TREN** at a maximum loading strain of 100%, and the cycle (blue) measured in 3 h after the 20th cycle under room temperature. (f) Residual strain plots of **aPTHF**_*x*_**-yTREN** as a function of cycle number, measured at a maximum loading strain of 100%. (g) Cyclic tensile curves of **aPTHF**_**2.9k**_**-1.3TREN**, measured at increased maximum loading strains from 20% to 200%.

The synthesis of **VU** polymer networks involved a cross-linking reaction between **aPTHF**_*x*_ and tris(2-aminoethyl)amine (**TREN**) initially in a drying oven at 60 °C overnight followed by postcuring the resulting film in a vacuum oven at 80 °C for 12 h. We synthesized several **VU** networks (**aPTHF**_*x*_**-yTREN**) by varying the *M*_n_ (*x*) of **aPTHF**_*x*_ and the molar ratio of amine to acetoacetate groups (**y**), with the latter parameter as 1.1, 1.3, or 1.5 by controlling the feeding ratio of **aPTHF**_*x*_ to **TREN** (Figure 2a; Table S2). The as-synthesized **aPTHF**_*x*_**-yTREN** series was first characterized by Fourier transform infrared (FTIR) spectroscopy. The FTIR spectra show that two peaks at 1716 and 1745 cm^–1^ ascribed to the acetoacetate groups of **aPTHF**_*x*_ initially appear upon the acetoacetylation of **PTHF**_*x*_ with excess **tBA** and then disappear following its cross-linking reaction with **TREN**. Concurrently, two new vibrational peaks at 1605 and 1645 cm^–1^ emerged, indicating the formation of **VU** linkages (Figure S14b–d). The intensity of the two newly emerged peaks increased upon increasing the value **y** in the FTIR spectra of the **aPTHF**_**2k**_**-yTREN** series, reflecting that more **VU** cross-links are formed (Figure S14e).

The chemical resistance of **aPTHF**_*x*_**-yTREN** was assessed by putting **aPTHF**_**2k**_**-1.3TREN** into various organic solvents (hexane, THF, ethanol, CHCl_3_, dioxane, and DMF), H_2_O, and 1 M NaOH at room temperature for 3 days (Figure S15). None of the samples dissolved; instead, they remained integral, indicating the robustness of the cross-linked networks and their strong resistance to these chemicals. Subsequently, we investigated the swelling ratio and gel fraction of the **aPTHF**_*x*_**-yTREN** networks by immersing them in ethanol for 24 h, with solvent renewal every 6 h (Figure 2b and Figure S16). As the *M*_n_ increased from 1 to 2.9 kg mol^–1^ in the **aPTHF**_*x*_**-1.3TREN** series, the swelling ratio gradually increased from 195% to 327%, while the gel fraction slightly decreased from 96% to 89% (Figure 2b). Increasing the value of **y** while keeping the *x* as 2k led to an obvious decrease in swelling ratio from 301% to 238% and no discernible variation in gel fraction, all around 92% (Figure S16). These results indicate that the cross-link density of the **aPTHF**_*x*_**-yTREN** series can be readily adjusted by controlling the *M*_n_ of **aPTHF**_*x*_ (i.e., *x*) and the feeding ratio of **TREN** to **aPTHF**_*x*_ (associated with **y**).

The thermal properties of the precursors **PTHF**_*x*_, **aPTHF**_*x*_, and the **aPTHF**_*x*_**-yTREN** series were evaluated by thermogravimetric analyses (TGA) and differential scanning calorimetry (DSC). TGA curves reveal that the decomposition temperature of **aPTHF**_*x*_**-1.3TREN** increases with the increase of value *x* (Figure S17a), while the thermal decomposition behavior within the **aPTHF**_**2k**_**-yTREN** series is comparable (Figure S17b). DSC heating traces of all measured samples, except **aPTHF**_**1k**_**-1.3TREN**, exhibited endothermic peaks at temperatures between 8 and 28 °C, which are associated with their melting temperatures *T*_m_ (Figure S18a–c). Evidently, the *T*_m_ and fusion enthalpy Δ*H*_c_ increased with the *M*_n_ (i.e., *x*) values of these samples. By comparing **PTHF**_*x*_ with the corresponding **aPTHF**_*x*_, the latter showed slight decreases in both *T*_m_ and Δ*H*_c_, from 21 °C and 74 J g^–1^, 27 °C and 88 J g^–1^, and 28 °C and 92 J g^–1^ of **PTHF**_*x*_ to 18 °C and 70 J g^–1^, 25 °C and 81 J g^–1^, 26 °C and 80 J g^–1^ of **aPTHF**_*x*_ with *x* = 1k, 2k, and 2.9k accordingly. Upon conversion of **aPTHF**_*x*_ into **aPTHF**_*x*_**-yTREN** networks, further decreases were noted. For example, **aPTHF**_**2.9k**_ exhibited a *T*_m_ of 26 °C and Δ*H*_c_ of 80 J g^–1^, and it dropped to 17 °C and 35 J g^–1^ of **aPTHF**_**2.9k**_**-1.3TREN**. Interestingly, **aPTHF**_**1k**_**-1.3TREN** did not show any endothermic peaks in the temperature of testing (−50 to 70 °C), indicating the absence of *T*_m_ (Figure S18a). Moreover, increasing the value of **y** and keeping the value of *x* the same led to decreases in *T*_m_ and Δ*H*_c_ as well (Figure S18d). The above results suggest that both the acetoacetylation of **PTHF**_*x*_ and the subsequent cross-linking reaction weaken the crystalline capabilities of resulting compounds through disrupting the highly ordered structure of **PTHF**_*x*_ or hindering the mobility of the polymer chains of **aPTHF**_*x*_, respectively. A higher cross-link density further restricts the chain mobility of the **aPTHF**_*x*_**-yTREN** networks.

We next studied the thermomechanical properties of **aPTHF**_*x*_**-yTREN** films by dynamic mechanical analysis (DMA). All DMA traces, except **aPTHF**_**1k**_**-1.3TREN**, displayed two distinct downtrends in the storage modulus (*E*’) as the temperature increased, accompanied by two shoulder peaks in the tan δ plots; one is around −18 °C and the other is between 22 and 30 °C (Figure 2c, Figure S19), which are attributed to the glass transition temperature (*T*_g_) and *T*_m_ of the films, respectively. Moreover, the second downtrend of *E*’ emerged at a higher temperature as the value *x* increased (Figure 2c) or the value **y** decreased (Figure S19). This indicates that a higher *T*_m_ is observed in the polymer featuring either a higher *M*_n_ of **aPTHF**_*x*_ or a lower **TREN** content, which mirrors the trend that we found in the DSC results (Figure S18). In the case of **aPTHF**_**1k**_**-1.3TREN**, the absence of the second downtrend in *E*’ and second shoulder peak in the tan δ plot marked no melting domain formed in the temperature range investigated (Figure 2c), previously confirmed by its DSC data as well (Figure S18a). Moreover, the DMA traces of these samples exhibited a rubbery plateau following the second downtrend in *E*’ (except **aPTHF**_**1k**_**-1.3TREN**, only exhibiting a rubbery plateau across the whole regime), corroborating the presence of cross-linking architecture in these polymers. According to the theory of rubber elasticity, cross-link density ν_*e*_ can be calculated by the following equation: ν_*e*_ = *E*’/[2(1 + ν)*RT*], where *E*’ is the storage modulus at *T*_m_ + 50 °C, ν is Poisson’s ratio (0.5), *T* is the temperature expressed in Kelvin, and *R* is the universal gas constant.^64^ The values of the rubbery plateau and ν_*e*_ for each film were proportional to the value **y** (Figure S19) and inversely proportional to the value *x* (Figure 2c). For example, the modulus of rubbery plateau and ν_*e*_ of **aPTHF**_**1k**_**-1.3TREN** were 6.1 MPa and 0.8 mol L^–1^, first decreased to 4.7 MPa and 0.6 mol L^–1^ of **aPTHF**_**2k**_**-1.3TREN**, and then down to 4.2 MPa and 0.5 mol L^–1^ of **aPTHF**_**2.9k**_**-1.3TREN**. As the value of **y** increased from 1.1 to 1.5, the modulus of rubbery plateau and ν_*e*_ increased from 3.9 MPa and 0.5 mol L^–1^ to 5.8 MPa and 0.8 mol L^–1^, respectively.

The mechanical properties of the **aPTHF**_*x*_**-yTREN** films were initially investigated by uniaxial tensile testing (Figure 2d, Figure S20). The stress–strain curves of all films displayed the typical behavior of elastomeric polymers, with the stress at break of 1.6–4.9 MPa and the strain at break of 50–390%. The influence of the *M*_n_ of building block on the tensile properties was reflected in Figure 2d. It revealed that both stress and strain at break continuously increased with *M*_n_ of **aPTHF**_*x*_. **aPTHF**_**1k**_**-1.3TREN** exhibited the lowest stress and strain at break (1.6 MPa, 46%), whereas **aPTHF**_**2.9k**_**-1.3TREN** had the highest stress and strain at break (4.8 MPa, 385%), representing 3- and 8-fold improvements, respectively. This suggests that the pronounced enhancement of mechanical properties can be achieved by using high *M*_n_ values of monomers in the polymer synthesis. In the **aPTHF**_**2k**_**-yTREN** series, increasing the **TREN** content, which corresponds to a higher cross-link density, consistently increased the stress at break. However, the strain at break initially rose and then declined (Figure S20). This is attributed to the increase of cross-link density resulting in an enhanced strength at the expense of the polymer’s chain movement.

The polymers’ elasticity and durability were analyzed by subjecting the **aPTHF**_*x*_**-yTREN** (*x* > 1k) films to cyclic tensile testing (Figure 2e, Figure S21). This experiment involved repeatedly stretching and releasing the films to a maximum strain of 100% at a loading/unloading rate of 50 mm min^–1^ for 20 cycles. No such experiment was made for **aPTHF**_**1k**_**-1.3TREN** due to its early fracture before the strain of 100% (Figure 2d). As shown in Figure 2e and Figure S21, all samples exhibited a substantial hysteresis loop in the first cycle followed by its shrinking from the second cycle onward. Concurrently, the residual strain after each cycle increased gradually until stabilizing around the 10th cycle (Figure 2f). For example, the residual strains for **aPTHF**_**2k**_**-yTREN** were 4%, 6%, and 5% after the first cycle with **y** = 1.1, 1.3, and 1.5 and slightly increased to 5%, 8%, and 7% by the fifth cycle, respectively. After an additional 5 cycles, the residual strain of **aPTHF**_**2k**_**-1.1TREN** remained stable while the other two slightly increased to 9% and 8%, respectively. 9% residual strain is also the maximum value seen in all the samples measured after being stretched for 20 successive cycles at a maximum strain of 100%, suggesting the excellent elasticity of **aPTHF**_*x*_**-yTREN** (*x* > 1k) films.

Through comparing the residual strains of **aPTHF**_**2k**_**-yTREN**, we observed that upon increasing the value of **y** (i.e., more **TREN** content), the residual strain exhibited the trend of first increasing and then decreasing, regardless of the cycle number (Figure 2f). When comparing **aPTHF**_**2k**_**-1.3TREN** with **aPTHF**_**2.9k**_**-1.3TREN** after the 10th cycle, the latter exhibited a lower residual strain (5% vs 9%), indicating that a longer chain length between cross-links enhances the elasticity of these polymer networks. Subsequently, **aPTHF**_**2.9k**_**-1.3TREN** was applied with a cyclic tensile test where the maximum loading strain was incrementally increased from 20% to 200% (Figure 2g). As shown in Figure 2g, the hysteresis loop expanded significantly with higher maximum loading strain, which is attributed to more chain movement and alignment under a larger tensile deformation. So did the residual strain, which saw increases upon increasing the tensile deformation (Figure S22). For example, **aPTHF**_**2.9k**_**-1.3TREN** showed almost no residual strain when stretched to 20%, but this increased to 5% at 100% loading strain and reached 11% at 200% loading strain. This phenomenon is because the cyclic tensile tests are carried out consecutively without rest between cycles, and the aligned and moved polymer chains do not have sufficient time for full recovery.

To explore whether the repetitive cyclic testing will cause permanent damage to the films (i.e., fatigue), we then relaxed the **aPTHF**_**2k**_**-1.3TREN** sample at room temperature for 3 h following the 20 successive cyclic tensile testing at a maximum loading strain of 100% (Figure 2e). The tensile curves showed that the initial hysteresis loop (black) shrunk significantly upon repetitively stretching and releasing **aPTHF**_**2k**_**-1.3TREN** for 20 cycles, and a subsequent 3 h relaxation time allowed for the resulting hysteresis loop (blue) to almost overlap with the initial one. These results confirm that this elastomer can resist external forces through polymer chain movement and/or alignment, and such responses are transient and can be alleviated with adequate relaxation time, highlighting the excellent durability of our elastomer.

### 3.3 Thermal Reprocessing and Acid-Assisted Recycling of the VU Polymer Networks

**Figure 3:**
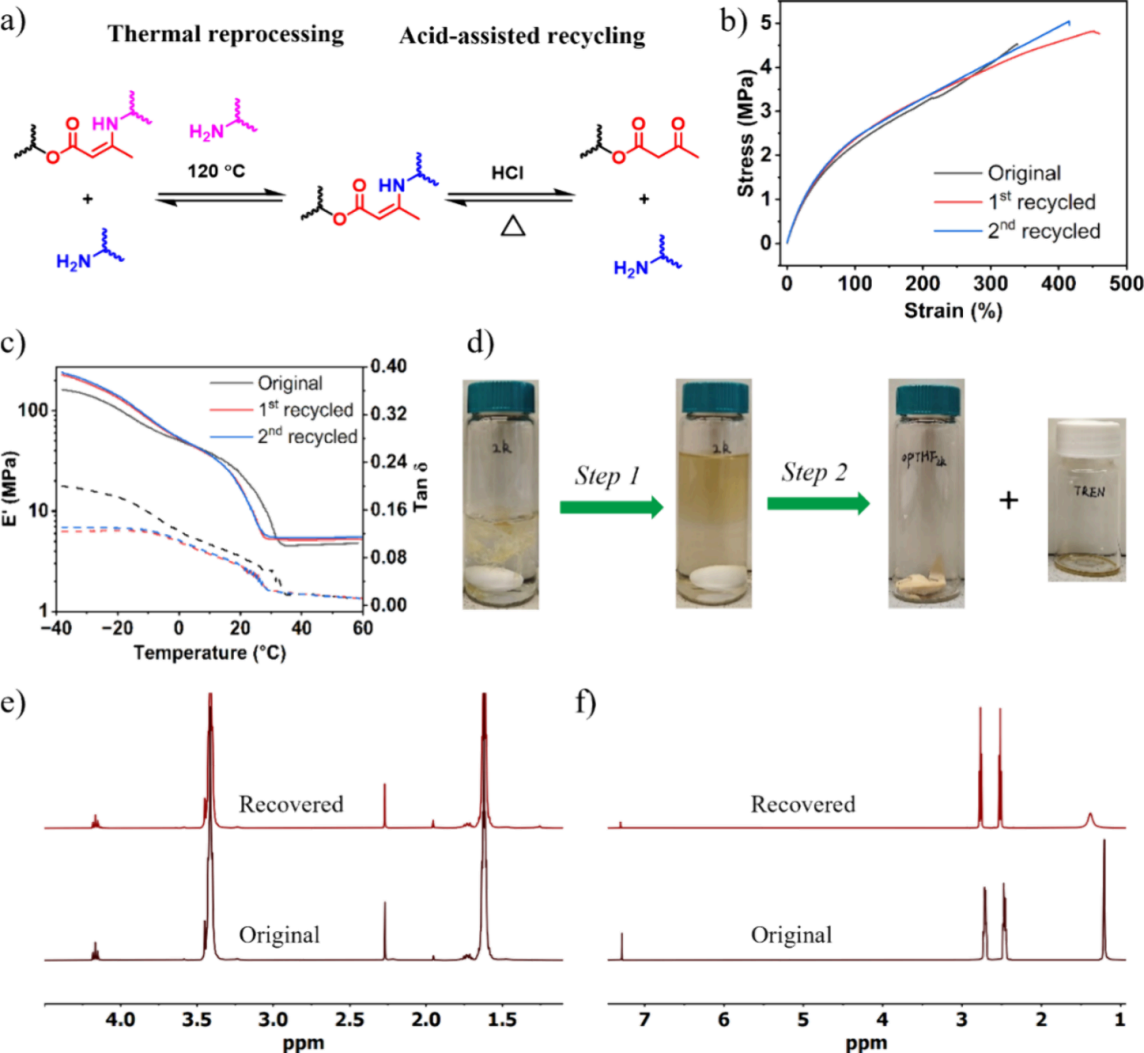
(a) The transamination reaction between an amine and a **VU** during thermal reprocessing (left), and the reversible reaction of a **VU** into an acetoacetate and an amine, with the dissociation triggered by HCl. (b) Stress–strain curves and (c) DMA traces of the original **aPTHF**_**2k**_**-1.3TREN**, and the reprocessed **aPTHF**_**2k**_**-1.3TREN** produced by compression-molding (*T* = 120 °C; *P* = 10 MPa; time = 20 min). (d) Photographs of **aPTHF**_**2k**_**-1.3TREN** shreds (0.5 g) initially dispersed in 15 mL of 1 M HCl (left), followed by the addition of 15 mL of diethyl ether to depolymerize the shreds (step 1), and the chemical separation of the depolymerized solution into **aPTHF**_**2k**_ and **TREN** (step 2). Comparison of the ^1^H NMR spectra of original (bottom) and recovered (top) **aPTHF**_**2k**_ (e) and **TREN** (f), with the recovery process shown in Figure 3d.

A key advantage of **VU**-cross-linked polymers over traditional thermosets lies in the dynamic nature of the **VU** linkages, which can participate in transamination reactions with auxiliary amines at elevated temperatures, as previously demonstrated by Du Prez et al.,^27,29^ or undergo dissociation via acid-assisted hydrolysis, as explored in this study. These properties enable **VU** polymer networks to be reused through two distinct paths: thermal reprocessing and chemical recycling. Thus, we presented the recycling of the **aPTHF**_*x*_**-yTREN** series by either (1) activating the exchange of **VU** cross-links with amine groups through compression-molding at 120 °C (Figure 3a; left) or (2) dissociating the **VU** linkages by HCl treatment (Figure 3a; right).

The flowability of the **aPTHF**_*x*_**-yTREN** films was initially examined by conducting stress relaxation experiments at temperatures between 80 and 130 °C prior to compression-molding (Figures S23 and S24; Table S3). The stress-relaxation curves reveal the occurrence of relaxation processes in all samples upon thermal treatment, with the stress dissipated effectively over time, and such dissipation is more pronounced at higher temperatures. For instance, at 80 °C, the relaxation times (τ), at which the stress is dissipated to 1/e of its initial value, were 1151, 1101, and 1456 s for **aPTHF**_*x*_**-1.3TREN** with *x* = 1k, 2k, 2.9k and decreased to 151, 240, and 371 s at 130 °C accordingly (Figure S23a–c). Moreover, a lower τ was also observed in the **aPTHF**_*x*_**-yTREN** sample prepared from either a lower *M*_n_ value of **aPTHF**_*x*_ (i.e., smaller **x**) or more **TREN** content (i.e., larger **y**). This is clearly supported by the decreases in τ from 441 s for **aPTHF**_**2.9k**_**-1.3TREN** to 215 s for **aPTHF**_**1k**_**-1.3TREN** (Figure S23a–c), or from 1353 s for **aPTHF**_**2k**_**-1.1TREN** to 213 s for **aPTHF**_**2k**_**-1.5TREN** (Figure S24a–c), all measured at 120 °C.

Linear fits of ln(τ) against 1/*T* (following the Arrhenius equation) allowed us to determine the activation energy (*E*_a_) for the stress relaxation (Figures S23d and S24d; Table S3). The *E*_a_ of the **aPTHF**_*x*_**-yTREN** samples decreased as the value of *x* increased or the value of **y** decreased. This is evidenced by the following orders of *E*_a_: 46 kJ mol^–1^ (*x* = **1k**, **y** = 1.3) > 28 kJ mol^–1^ (*x* = **2k**, **y** = 1.3) > 26 kJ mol^–1^ (*x* = **2.9k**, **y** = 1.3) (Figure S23d); 40 kJ mol^–1^ (*x* = 2k, **y = 1.5**) > 28 kJ mol^–1^ (*x* = 2k, **y = 1.3**) > 19 kJ mol^–1^ (*x* = 2k, **y = 1.1**) (Figure S24d). A higher *E*_a_ indicates a stronger temperature dependence of stress relaxation, in other words, a slight variation in temperature can significantly affect the relaxation time of the polymer that possesses a higher *E*_a_ for its stress relaxation.^65^ Together with the trends observed in relaxation times of the networks, these results suggest that shorter chain length between **VU** linkages and/or more free amine groups presented in the networks accelerate the stress relaxation process and afford the **aPTHF**_*x*_**-yTREN** polymers with a more sensitive stress-relaxation response to processing temperature. Therefore, modulation of these two parameters allows us to access **VU** polymers with a wide spectrum of stress-relaxation abilities. This detailed information points to guiding the (re)processing of **aPTHF**_*x*_**-yTREN** networks in a more time- and energy-efficient manner.

We next set out to thermally reprocess **aPTHF**_*x*_**-1.3TREN** (*x* = 1k, 2k, and 2.9k) polymers by first cutting the films into small pieces followed by compression-molding them at 120 °C under a pressure of 10 MPa for 20 min. The treatment produced three homogeneous films (Figure S25). The chemical structures, mechanical, and swelling properties of the reprocessed films were measured by FTIR, tensile testing, and swelling experiments and then used to compare with those of the pristine films (Figure 3b–c, Figures S26–229). Intriguingly, there are almost no discernible differences in the FTIR spectra of the pristine and thermally reprocessed polymers, reflecting no apparent damage to the chemical structures of these reprocessed films (Figure S28). Moreover, taking **aPTHF**_**2k**_**-1.3TREN** as the example, the reprocessed samples showed slight increases in both stress and strain at break in the stress–strain curves (Figure 3b) and exhibited a slight increase in *E*’ of the rubbery regime and a decrease of *T*_m_ in the DMA traces (Figure 3c). These minor changes are attributed to the conversion of some unreacted amines and acetoacetates in the pristine material into **VU** linkages during thermal reprocessing, resulting in a film with a marginally higher cross-link density. This is further corroborated by the observations of the slight decrease of swelling ratio and the minor increase of gel fraction in the thermally reprocessed **aPTHF**_**2k**_**-1.3TREN** as compared to the pristine one (Figure S29).

As established above by model studies, we expect the **aPTHF**_*x*_**-1.3TREN** thermosets to be depolymerizable upon acidic treatment in the presence of a two-phase mixture solution. To test this, 0.5 g of **aPTHF**_*x*_**-1.3TREN** (*x* = 1, 2, or 2.9k) shreds, collected after the tensile testing and DMA measurements, was first added to 15 mL of 1 M HCl. The **aPTHF**_**1k**_**-1.3TREN** polymer got disintegrated in the solution over time and eventually formed into a slurry after 18 h, while **aPTHF**_**2k**_**-1.3TREN** was transformed into a paste-like object and **aPTHF**_**2.9k**_**-1.3TREN** maintained a good dimension (Figure 3d, Figure S30). These observations reflect that the polymer prepared by a high *M*_n_ of **aPTHF**_*x*_ demonstrates increased resistance to strong acids, probably due to the enhanced hydrophobicity. To fully depolymerize these thermosets, 15 mL of diethyl ether was further added to the mixtures and reacted for another 24 h. All three cases afforded clear mixture solutions, with the diethyl ether suspended above 1 M HCl, indicating the successful depolymerizations.

We next attempted the recovery of **aPTHF**_*x*_ (*x* = 1k, 2k, or 2.9k) and **TREN**. **aPTHF**_*x*_ should exist in the diethyl ether phase. Thus, separation of diethyl ether solution with 1 M HCl by a separation funnel followed by washing the ether solution with water, and the subsequent evaporation of diethyl ether afforded **aPTHF**_*x*_ in a yield of 90%–98%. The separated HCl phase after depolymerization of all **aPTHF**_*x*_**-1.3TREN** samples was combined together for the recovery of **TREN**. The combined 1 M HCl solution was washed with diethyl ether three times and then neutralized by 1 M NaOH. Evaporation of the neutralized solution followed by extraction with CHCl_3_ and subsequent removal of the CHCl_3_ afforded **TREN** in an 86% yield. The chemical structures of the recovered **aPTHF**_*x*_ and **TREN** were assessed by ^1^H NMR spectroscopy (Figure 3e,f, Figures S31 and S32), with comparisons of ^1^H NMR spectra made between the pristine and recovered **aPTHF**_*x*_ and **TREN** (Figure 3e,f, Figures S31 and S32), which do not show detectable impurities and confirm good recovery of all the components.

The recovered **aPTHF**_**2k**_ and **TREN** were then used to repolymerize into a new **aPTHF**_**2k**_**-1.3TREN** film following the same procedures as those of preparing a pristine one. The repolymerized film was then characterized by FTIR spectroscopy (Figure S33), tensile testing and DMA (Figure S34), and swelling experiments (Figure S35), with the results compared with those of pristine **aPTHF**_**2k**_**-1.3TREN**. No distinct variations were observed in the measurements of the two samples, demonstrating the effectiveness of such an acid-assisted recycling method in the reuse of **VU** thermosets.

### 3.4 Synthesis, Sensing Performance, and Chemical Recycling of the Strain Sensor

Having confirmed the excellent elasticity and depolymerization capability of **aPTHF**_*x*_**-yTREN**, we realize that our thermosetting films could serve as promising recyclable substrates for strain sensors. To substantiate this, in the final part of our work, we conducted a case study by first synthesizing a strain sensor out of **aPTHF**_**2k**_**-1.3TREN** and multiwalled carbon nanotubes (**MCNs**) followed by investigating its sensing capability and then chemical recyclability. The synthesis of stain sensor involved the initial incorporation of 5 wt % **MCNs** in the reaction mixture of **aPTHF**_**2k**_ and **TREN** with a 1.3:1 molar ratio of amines to acetoacetates. Subsequent thermal treatment at 80 °C and compression-molding at 120 °C resulted in a black flat film, labeled **VU/5%MCNs**, with a thickness of ca. 0.2 mm.

**Figure 4:**
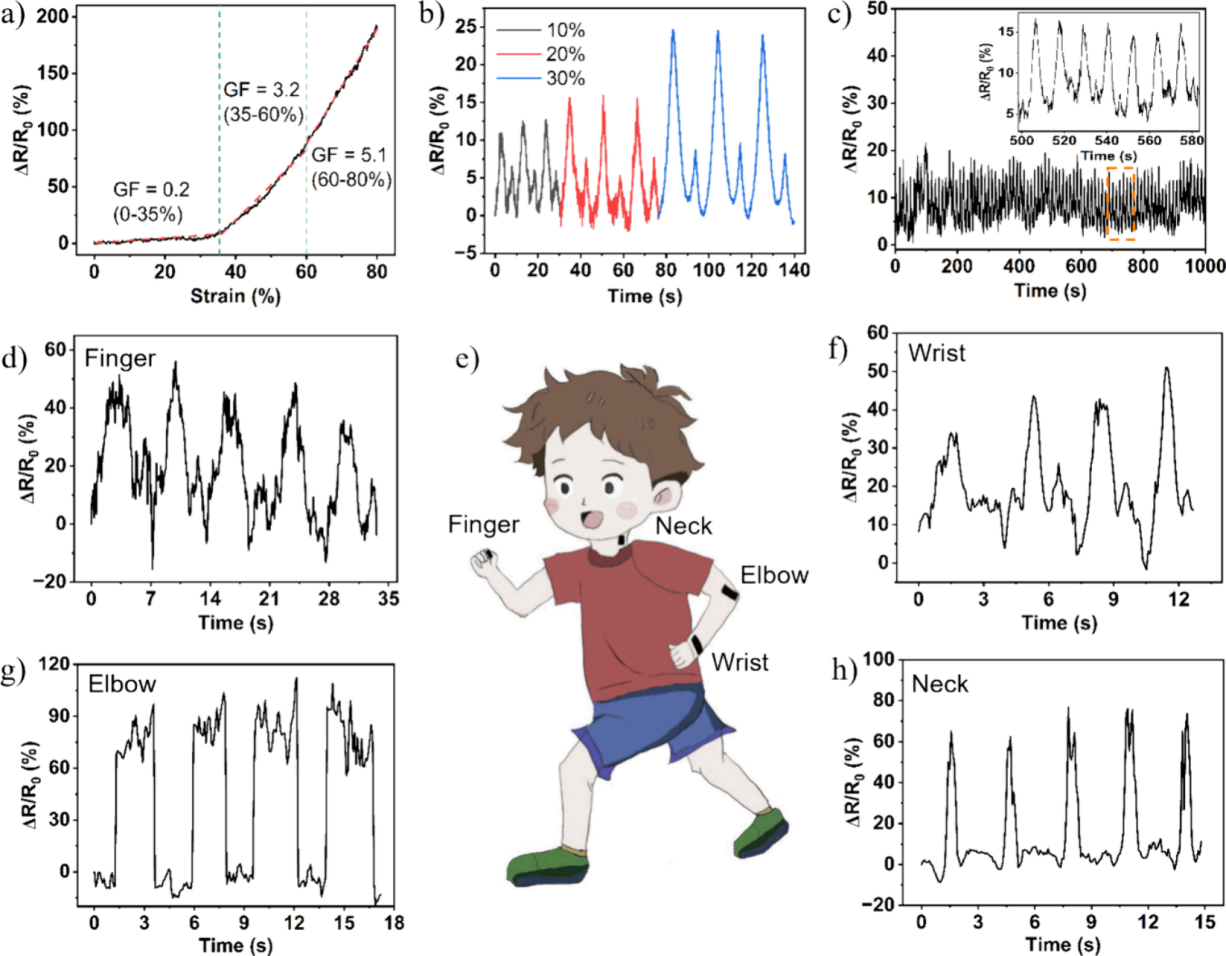
(a) Relative resistance variation (Δ*R*/*R*_0_) of the **VU/5%MCNs** strain sensor as a function of tensile strain. (b) Δ*R*/*R*_0_ of the strain sensor under 3 consecutive loading/unloading cycles, with the maximum loading strain of 10% (black), 20% (red), and 30% (blue). (c) Sensing stability of the strain sensor during 100 consecutive cycles of stretching–releasing with the maximum loading strain of 20%. Demonstration of the **VU/5%MCNs** strain sensor monitoring various motions including the bending and stretching movements of (d) finger, (f) wrist, and (g) elbow, and (h) neck craning and returning, with the sensor attached on the corresponding body parts shown in panel (e).

The **VU/5%MCNs** sensor (25 × 5 × 0.2 mm; *l* × *w* × *t*) thus produced was subjected to uniaxial tensile testing at a loading rate of 10 mm min^–1^, while being connected to a source meter (Keithley 2450, US) to monitor the real-time resistance (*R*) at a constant current of 100 μA. The resistance variation (Δ*R = R* – *R*_0_) was normalized by the initial resistance (*R*_0_) to calculate the relative resistance variation (Δ*R*/*R*_0_), which was then plotted against tensile strain (Figure 4a). Upon increasing the tensile strain, the value of Δ*R*/*R*_0_ exhibited three responsive behaviors; the first one in the strain range 0–35% had a slope of 0.2, the second one (35–60%) had a slope of 3.2, and the third one was in the range 60–80% with a slope of 5.1. The slope represents gauge factor (GF), related to the sensitivity of the strain sensor to applied tensile strain. Moreover, by applying cyclic tensile testing on the sensor under 10%, 20%, and 30% at a loading/unloading rate of 10 mm min^–1^ (Figure 4b), the Δ*R*/*R*_0_ signals exhibited stable waveforms with increased values observed at the larger applied deformations. After 100 consecutive stretching and releasing cycles at 20% strain at a rate of 10 mm min^–1^, the Δ*R*/*R*_0_ signals remained consistent, indicating the good robustness of our sensor.

Based on the sensing capabilities of **VU/5%MCNs** for various deformations, the sensor was then affixed to different parts of the body (as indicated in Figure 4e), including the finger, wrist, elbow, and neck (Figure 4d–h). The Δ*R*/*R*_0_ signals exhibited distinct variations in response to different body movements, characterized by unique waveforms and intensities. Usually, the value of Δ*R*/*R*_0_ increased during the motion (e.g., finger, wrist, and elbow flexion, neck craning), and then reverted to its initial state upon the release of such a motion (Figure 4d–h).

**Figure 5:**
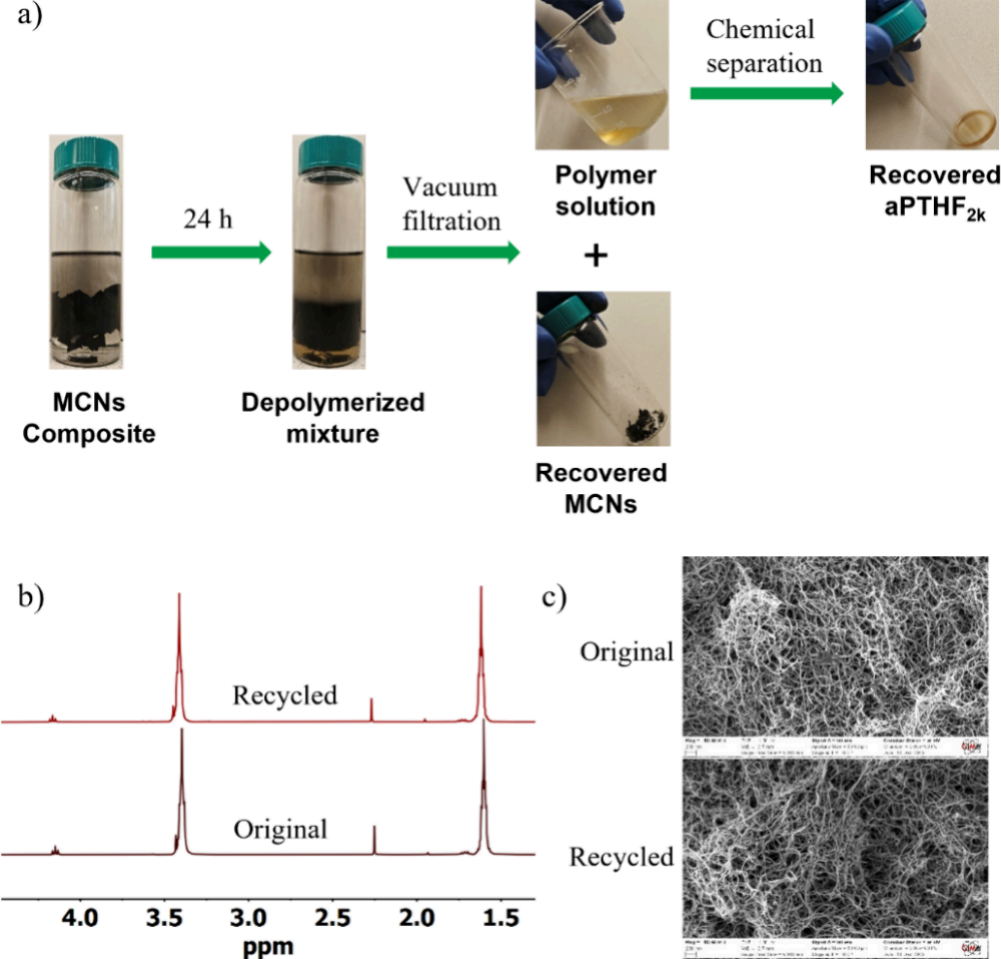
(a) Photographs showing the depolymerization of the **aPTHF**_**2k**_**-1.3TREN/MCNs** composite (0.5 g) by a mixture solution comprising 10 mL of 1 M HCl and 10 mL of diethyl ether for 24 h, the separation of **MCNs** and depolymerized polymer solution by vacuum filtration followed by the purification of the depolymerized solution to recover **aPTHF**_**2k**_. Comparisons of the (b) ^1^H NMR spectra and (c) SEM images of original **aPTHF**_**2k**_ and **aPTHF**_**2k**_ recovered from the depolymerization of **MCNs**-incorporated **VU** composite.

Finally, we investigated the recycling of the **VU/5%MCNs** strain sensor by immersing 0.5 g of the sensor composite in a 20 mL diethyl ether–1 M HCl mixture solution (1:1, v/v) for 24 h (Figure 5a). The sensor depolymerized over time, and the **MCNs** accumulated in the 1 M HCl phase. Vacuum filtration of the depolymerized mixture allowed for separation of **MCNs** with the polymer solution (Figure 5a). The diethyl ether phase of the mixture solution was collected by a separation funnel, washed with pure water three times, and then dried to recover **aPTHF**_**2k**_ in a high yield (95%) and in high purity (Figure 5b). Moreover, analysis of **MCNs** before and after the recycling with scanning electron microscopy (SEM) and energy-dispersive X-ray analysis (EDX) provided clear evidence of the unperturbed morphology and chemical structure of the recovered **MCNs** (Figure 5c, Figure S36).

## 4 Conclusions

In summary, we have presented a new approach to investigating the kinetics of the acid-catalyzed hydrolysis of hydrophobic **VU**s by using a heterogeneous system comprising two immiscible solvents—HCl and CDCl_3_. The results show that the hydrolysis rate of the **VU** linkages has a strong dependence on the reaction temperature and HCl concentration, with the higher values of each leading to increased hydrolysis rates. This approach is poised to study the kinetic and thermodynamic aspects of the hydrolysis for other hydrophobic and hydrolyzable chemical motifs, including but not limited to imines, boronate esters, 1,3-dioxolane, and boroxines. These chemical motifs have shown promises in the synthesis of chemically stable and yet hydrolyzable polymers. By elucidating the mechanistic details of their hydrolysis, we anticipate that it will further catalyze the application and recycling of these recyclable polymers in our daily life by optimizing the technologically relevant chemical engineering process.

Regarding the material’s part, we systematically investigated the structure–property relationships of the **VU** elastomers, obtained by reacting polytetrahydrofuran bisacetoacetate (**aPTHF**) and tris(2-aminoethyl)amine (**TREN**). The key properties, including thermal, mechanical, and thermomechanical behaviors, stress relaxation, and elasticity, could be flexibly adjusted by modulating the molecular weight of **aPTHF** and the network cross-link density. The **VU** elastomers are closed-loop recyclable upon acidic treatment at room temperature, allowing for the good recovery of **aPTHF** (>90%) and **TREN** (86%). Finally, we explored the viability of this **VU** elastomer in the synthesis of strain sensors by incorporating multiwall carbon nanotubes (**MCN**s) into the reaction mixture of **aPTHF** and **TREN**. The resulting composite exhibits robust sensing performance, effectively detecting a range of body motions. Most importantly, the sensor is also closed-loop recyclable upon acidic treatment for 24 h, allowing for the recovery of **aPTHF** in 95% yield and **MCN**s in high quality. We therefore envision that these elastomers could significantly contribute to the development of next-generation, sustainable wearable sensors.

## Supporting Information Available

Instrumentation; synthesis and characterization; synthesis of polytetrahydrofuran bisacetoacetate (aPTHF_x_); synthesis of bis-(butyl acetoacetate)-terminated polytetrahydrofuran (aPTHF_2k_-Btl); kinetic measurements of the dissociation of aPTHF_2k_-Btl by ^1^H NMR spectroscopy; determination of the activation energy for the dissociation of aPTHF_2k_-Btl with 1M HCl; synthesis of vinylogous urethane polymer networks; activation energy for aPTHF_x_-yTREN networks; depolymerization of aPTHF_x_-1.3TREN and subsequent recovery of starting materials; synthesis, depolymerization, and recovery of starting materials of strain sensor (PDF)
