# Supplementary material for: Structure–Property Relationships of Elastomeric Vinylogous Urethane Thermosets and Their Application as Closed-Loop Recyclable Strain Sensors
Source: Macromolecules. 2025 Feb 5;58(4):1923–34. doi: 10.1021/acs.macromol.4c03256 (PMC11866918; doi:10.1021/acs.macromol.4c03256)
Supplement: Supplementary file 1 — ma4c03256_si_001.pdf [file ma4c03256_si_001.pdf]

Support Information

for

Structure-Property Relationships of Elastomeric Vinylogous Urethane Thermosets and Their Application as Closed-Loop Recyclable Strain Sensors

Youwei Ma,[1*] Francesco Stellacci[1,2]

1. Institute of Materials, École Polytechnique Fédérale de Lausanne (EPFL) Lausanne 1015, Switzerland

2. Institute of Bioengineering, École Polytechnique Fédérale de Lausanne (EPFL) Lausanne 1015, Switzerland

*Correspondence: Y. Ma, youwei.ma@epfl.ch

Contents

Instrumentation S2

Synthesis and Characterization S4

Synthesis of polytetrahydrofuran bisacetoacetate (**aPTHF$_x$**) S4

Synthesis of bis-(butyl acetoacetate)-terminated polytetrahydrofuran (**aPTHF$_{2k}$-Btl**) S8

Kinetic measurements of the dissociation of **aPTHF$_{2k}$-Btl** by ^{1}H NMR spectroscopy S11

Determination of the activation energy for the dissociation of **aPTHF$_{2k}$-Btl** with 1M HCl S16

Synthesis of vinylogous urethane polymer networks S19

Activation energy for **aPTHF$_x$-yTREN** networks S25

Depolymerization of **aPTHF$_x$-1.3TREN** and subsequent recovery of starting materials S32

Synthesis, depolymerization, and recovery of starting materials of strain sensor S37

Reference S38

Instrumentation

Nuclear Magnetic Resonance (NMR) spectroscopy. NMR spectroscopy was carried out at 297.2 K on a Bruker Avance DPX 400 spectrometer at frequencies of 400.19 MHz for 1H nuclei. Spectra were calibrated to the residual solvent peak of $CDCl_3$ (7.26 ppm 1H NMR), D_2O (4.79 ppm, 1H NMR). Data were evaluated with the MestReNova software suite (v 12.0) and all chemical shifts (δ) are reported in parts per million (ppm) relative to tetramethylsilane with coupling constant (J) in Hz (multiplicity: s = singlet, d = doublet, t = triplet, q = quartet, m = multiplet, dd = double doublet).

Fourier Transform Infrared (FTIR) Spectroscopy. FTIR was carried out on a Bruker Tensor 27 spectrometer. Samples were analyzed in attenuated total reflectance (ATR) mode using a diamond crystal.

Thermogravimetric Analyses (TGA) were performed with a Mettler-Toledo TGA/DSC 1 Stare System. The temperature ranged from 25 °C to 900 °C with a heating rate of 10 °C min^{-1}. Tests were carried out under nitrogen with a flow rate of 40 mL min^{-1}. TGA results were analyzed using the STARe Evaluation software.

Differential Scanning Calorimetry (DSC) measurements were performed under a nitrogen atmosphere using a Mettler-Toledo STAR system operating at a heating/cooling rate of 10 °C min^{-1} in the temperature range of -80 to 70 °C using a sample mass of ca. 5 mg. The melting temperature, T_m, is reported based on the minimum of the major endothermic melting peak.

Dynamic Mechanical Analyses (DMA) were performed on the TA Instrument Model Q800 in tension mode. The temperature ranged from -40 to 60 °C with a heating rate of 3 °C min^{-1}, a frequency of 1Hz, and an amplitude of 0.5% strain.

Tensile testing. If not indicated, stress-strain measurements were carried out at 50 mm min^{-1} with a Zwick/Roell Z010 tensile tester equipped with a 50 N load cell. Uniaxial tensile tests were carried out with dog-bone-shaped samples with dimensions of 40 × 5 × 0.2 mm (length × width × thickness) that were cut from the solution-casted films according to ASTM D1708. The testing procedure followed the standard method described in ASTM D1708. Cyclic tensile tests were also carried out on the Zwick/Roell Z010 machine with a loading/unloading rate of 50 mm min^{-1}.

Solubility experiment. The weighed samples ($M_{initial}$) were immersed in ethanol and shaken in a shaking bed for 24 h. The ethanol was changed every 6 h. The solvent was carefully removed with a syringe, and the surface was wiped with a tissue after swelling, after which the samples were weighed ($M_{swelling}$). The samples were dried under vacuum at 80 °C for 24 h to ensure complete removal of the solvent from the material. After drying, the samples were weighed again (M_{drying}). The swelling ratio was defined as $M_{swelling}/M_{initial}$, whereas the gel fraction was defined as $M_{drying}/M_{initial}$.

Compression testing. Compression tests were performed with a Carver 3851-0 press. The samples were cut into small pieces using scissors. The pieces were piled up and placed between two sheets of Teflon, compressed between two plates of steel at a distance of 0.2 mm, applying a pressure of 10 MPa for 20 min at 120 °C.

Scanning Electron Microscopy (SEM) and Energy Dispersive X-Ray Analysis (EDX). The morphology and chemical composition of the multi-walled carbon nanotubes (**MCNs**) before and after the chemical recycling of the polymer composites were analyzed by SEM equipped with EDX on a Magellan 400 manufactured by FEI.

Electrical conductivity. All of the relative resistance changes were measured using a Keithley 2450 source meter. For the stretching test, the polymeric strain sensor was fixed on Zwick/Roell Z010 tensile tester to apply different strains. For body motion detections, the strain sensor was attached on human skin at different body parts (including index finger knuckle, wrist joint dorsum, elbow joint, front neck) by using an adhesive medical tape. Various body motions were performed.

Synthesis and Characterization

Synthesis of polytetrahydrofuran bisacetoacetate (aPTHF$_x$)

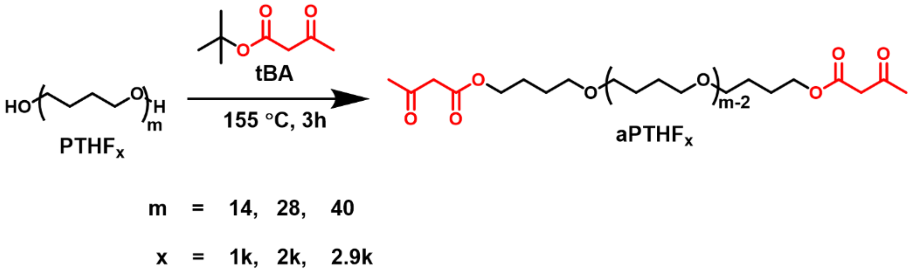

Figure S1. Synthesis of **aPTHF$_x$**.

Commercial **PTHF$_x$** (10 g, **x** = 1k for 1000 g mol^{-1}, **x** = 2k for 2000 g mol^{-1}, **x** = 2.9k for 2900 g mol^{-1}) was weighed and added into a 50 mL single-necked flask, together with a stirring bar. The flask was equipped with a condenser and a Dean-Stark apparatus to remove the *tert*-butanol generated during the reaction. Then, *tert*-butyl acetoacetate (**tBA**, 10 equiv.) was added in one portion with a pipette, and the resulting mixture was heated at 155 °C for 3 h. The unreated **tBA** was removed by vacuum distillation at 100 °C for 12 h to afford polytetrahydrofuran bisacetoacetate (**aPTHF$_x$**). The extent of end-group functionalization was determined by integration of the ^{1}H NMR signals in Figures S2-4 following the equation:

Conversion rate = (m × 2 - 4)/Integration of peak *d* × 100

aPTHF$_{1k}$: yield 95%, 11.1 g; ^{1}H NMR (400 MHz, CDCl$_3$) δ 4.10 (t, *J* = 6.5 Hz, 2H), 3.38 (s, 2H), 3.38 – 3.28 (m, 24H), 2.20 (s, 3H), 1.55 (dq, *J* = 6.0, 3.1 Hz, 27H).
The conversion was estimated from the ^{1}H NMR spectrum in Figure S2: (14 × 2 - 4)/24.5 × 100 = 98%.

aPTHF$_{2k}$: yield 98%, 10.5 g; ^{1}H NMR (400 MHz, CDCl$_3$) δ 4.15 (t, *J* = 6.5 Hz, 2H), 3.43 (s, 2H), 3.40 (h, *J* = 3.1 Hz, 52H), 2.25 (s, 3H), 1.61 (h, *J* = 3.1 Hz, 54H).

The conversion was estimated from the ^{1}H NMR spectrum in Figure S3: (28 × 2 - 4)/52.1 × 100 = 100%.

aPTHF$_{2.9k}$: yield 91%, 9.9 g; ^{1}H NMR (400 MHz, CDCl$_3$) δ 4.10 (t, *J* = 6.6 Hz, 2H), 3.38 (s, 2H), 3.34 (t, *J* = 5.4 Hz, 78H), 2.20 (s, 3H), 1.55 (p, *J* = 3.1 Hz, 80H).

The conversion was estimated from the ^{1}H NMR spectrum in Figure S4: (40 × 2 - 4)/78 × 100 = 97%.

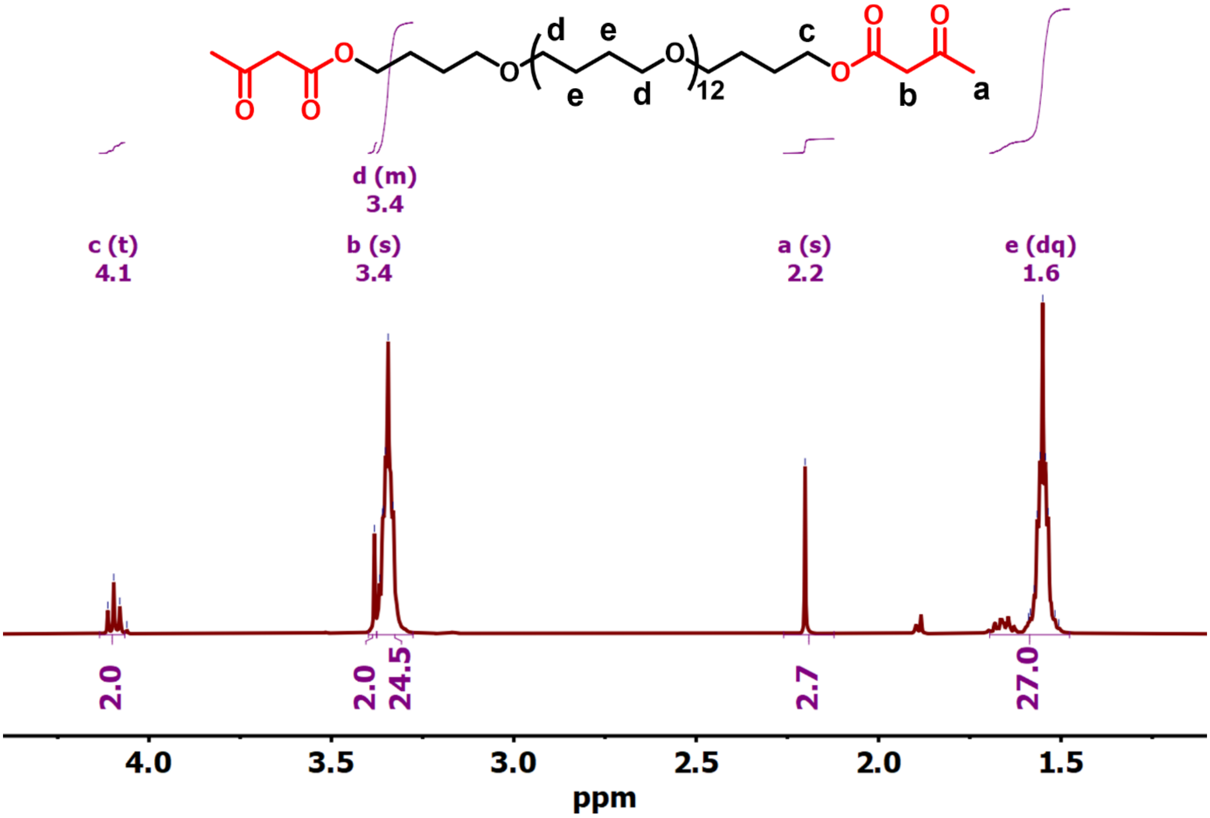

Figure S2. ^{1}H NMR spectrum ($CDCl_3$, 400 MHz) of **aPTHF$_{1k}$**.

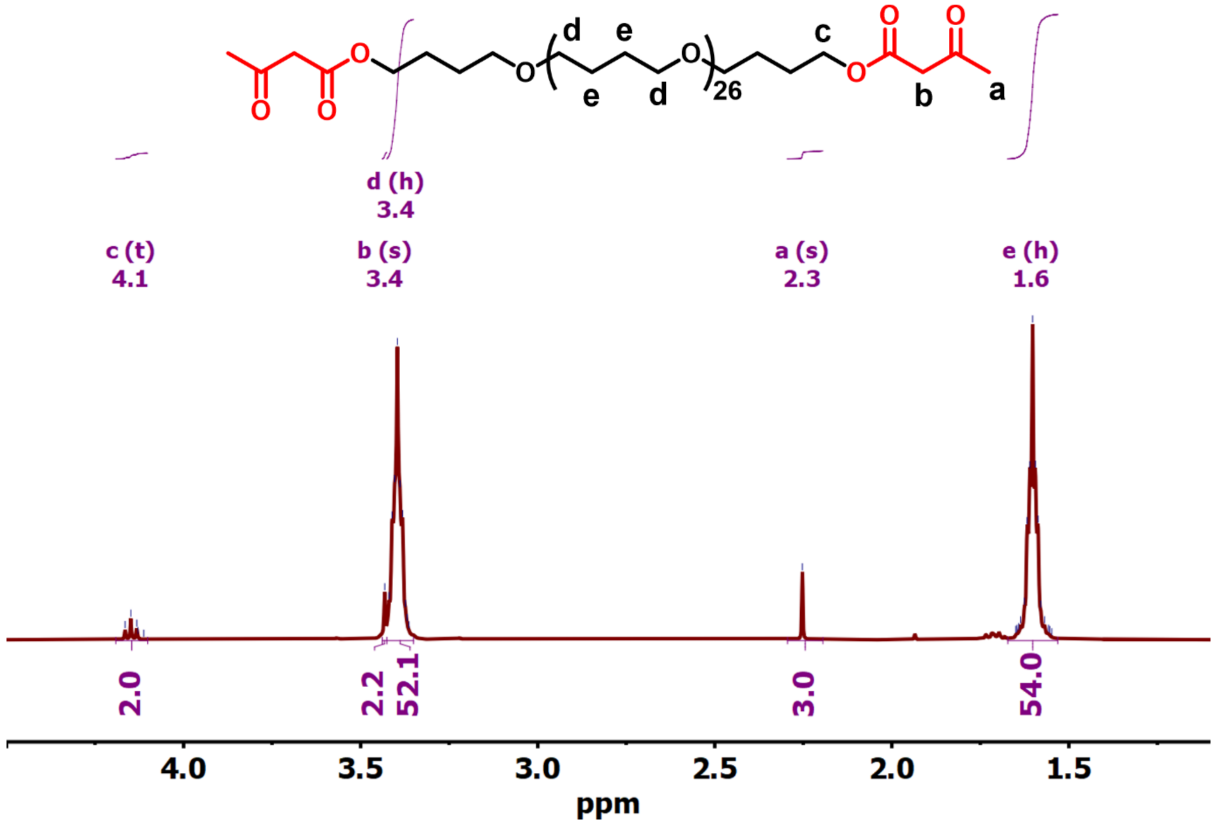

Figure S3. ^{1}H NMR spectrum ($CDCl_3$, 400 MHz) of **aPTHF$_{2k}$**.

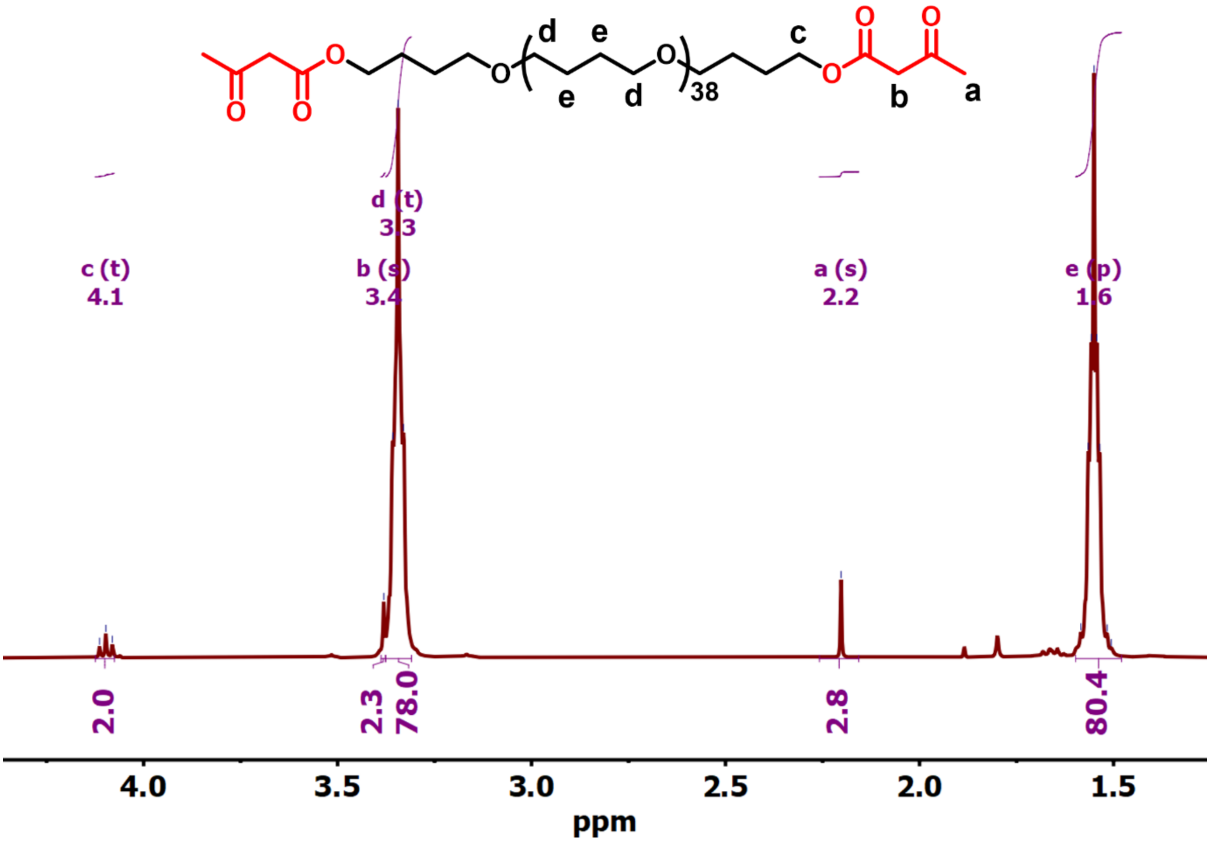

Figure S4. ^{1}H NMR spectrum ($CDCl_3$, 400 MHz) of **aPTHF$_{2.9k}$**.

Synthesis of bis-(butyl acetoacetate)-terminated polytetrahydrofuran (aPTHF$_{2k}$-Btl)

As shown in Figure S5, in a 25 mL two-necked flask equipped with a reflux condenser and a magnetic stir bar, butylamine (**Btl**; 0.73 g, 10 mmol) was combined with **aPTHF$_{2k}$** (2.2 g, 1 mmol) in 10 mL of THF. The reaction mixture was heated to 60 °C for 24 h while stirring. The excess of **Btl** and THF was removed by rotary evaporation, followed by subsequent vacuum drying at 70 °C for 12 h. Bis-(butyl vinylogous urethane)-terminated polytetrahydrofuran (**aPTHF$_{2k}$-Btl**) was obtained as yellowish liquid (2.2 g; yield 95%).

^{1}H NMR (400 MHz, $CDCl_3$) δ 8.71 – 8.22 (m, 1H), 4.41 (s, 1H), 4.04 (t, *J* = 6.2 Hz, 2H), 3.41 (h, *J* = 3.1 Hz, 52H), 3.22 (d, *J* = 9.8 Hz, 2H), 1.94 (d, *J* = 23.8 Hz, 3H), 1.81 – 1.46 (m, 56H), 1.40 (h, *J* = 7.2 Hz, 2H), 0.93 (t, *J* = 7.3 Hz, 3H).

^{13}C NMR (101 MHz, $CDCl_3$) δ 170.67, 163.12, 81.67, 70.78, 62.19, 42.71, 32.41, 29.57, 26.68, 19.99, 13.75.

The ^{1}H NMR and ^{13}C NMR spectra are shown in Figures S6 and S7, respectively.

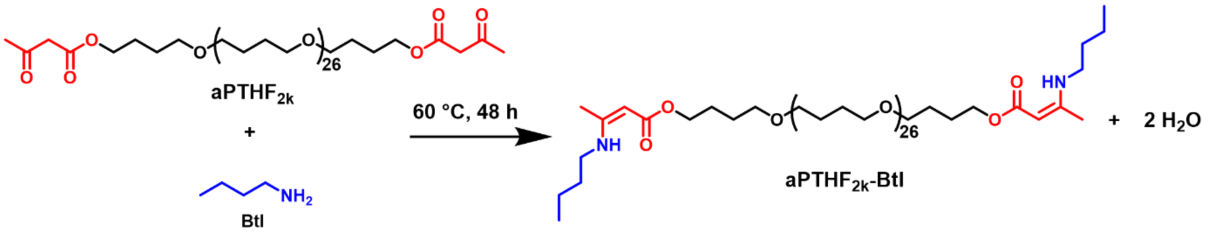

Figure S5. Synthesis of **aPTHF$_{2k}$-Btl**.

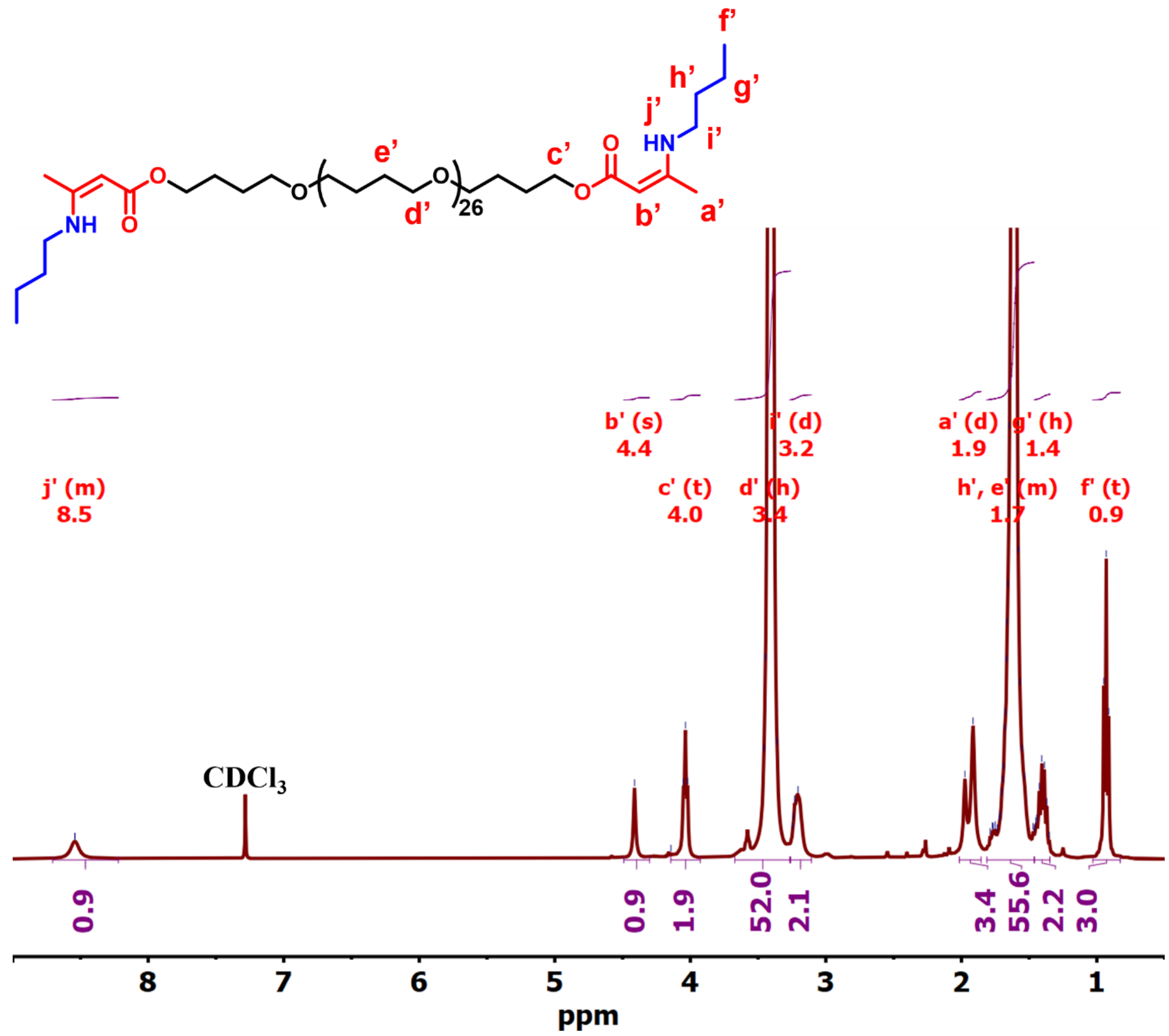

Figure S6. ^{1}H NMR spectrum ($CDCl_3$, 400 MHz) of **aPTHF$_{2k}$-Btl**.

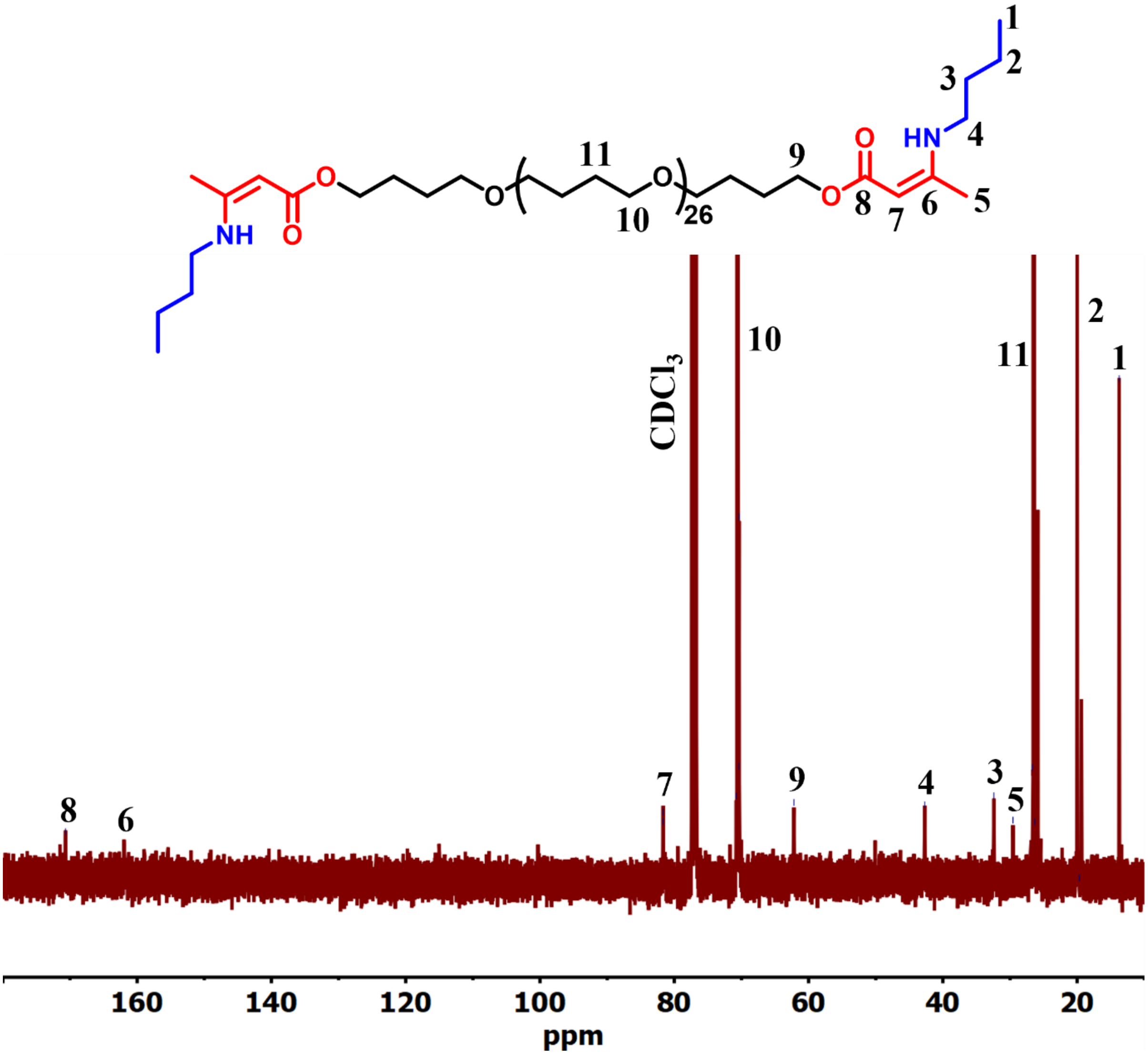

Figure S7. ^{13}C NMR spectrum ($CDCl_3$, 101 MHz) of **aPTHF$_{2k}$-Btl**.

Kinetic measurements of the dissociation of aPTHF$_{2k}$-Btl by ^{1}H NMR spectroscopy

aPTHF$_{2k}$-Btl features high hydrophobicity and can not dissolve in water. A two-phase mixture solution comprising different amounts of 1M HCl (upper phase) and 4 mL of $CDCl_3$ (bottom phase) was used to investigate the influence of HCl volume on the dissociation of **aPTHF$_{2k}$-Btl** (Figure S8a). Specifically, 200 mg of **aPTHF$_{2k}$-Btl** was first dissolved in 4 mL of $CDCl_3$ in a 20 mL vial. Different amounts of 1M HCl (1 mL, 2 mL, and 4 mL) were then added and suspended above the $CDCl_3$ solution. The resulting mixture was kept at 20 °C while stirring at a rate of 400 rpm min^{-1}. After different reaction time intervals, 0.5 mL aliquots of the $CDCl_3$ solution phase were taken out with a syringe and transferred to an NMR tube. ^{1}H NMR spectra of these solutions were measured (Figures S8c-e) and the data were used to establish the reaction kinetics. The proton assignment before and after the dissociation is shown in Figure S8(b).

The fraction of **aPTHF$_{2k}$** was calculated by integration of the ^{1}H NMR signals, according to the equation:

Fraction **aPTHF$_{2k}$** = [**aPTHF$_{2k}$**]/([**aPTHF$_{2k}$**]+[**aPTHF$_{2k}$-Btl**]) = Integral of proton *c*/(Integral of proton *c* + Integral of proton *c*').

The extent of conversion in different amounts of 1M HCl is estimated by the fraction of **aPTHF$_{2k}$** (calculated by integration of the ^{1}H NMR signals in Figures S8c-e) and plotted in Figure 1(d).

The impact of stirring rate on the hydrolysis of **aPTHF$_{2k}$-Btl** was investigated in the same setup using a different stirring rate of 100, 400, and 800 rpm min^{-1} (Figure S9a). 200 mg of **aPTHF$_{2k}$-Btl** was first dissolved in the mixture of 4 mL $CDCl_3$ and 2 mL 1 M HCl in a 20 mL vial. The resulting solution was kept at 20 °C while stirring at a rate of 100, 400, and 800 rpm min^{-1}. After different reaction time intervals, 0.5 mL aliquots of the $CDCl_3$ solution phase were taken out with a syringe and transferred to an NMR tube. ^{1}H NMR spectra of these solutions were measured (Figures S9c-e) and the data were used to establish the reaction kinetics. The proton assignment before and after the dissociation is shown in Figure S9(b).

The extent of conversion measured at the different stirring rate is estimated by the fraction of **aPTHF$_{2k}$** (calculated by integration of the ^{1}H NMR signals in Figures S9c-e) and plotted in Figure S9(f).

The influence of HCl concentration (0.1 M, 0.5 M, and 1 M) on the dissociation of **aPTHF$_{2k}$-Btl** was investigated using the same setup (Figure S10a). First, 200 mg of **aPTHF$_{2k}$-Btl** was dissolved in 4 mL of $CDCl_3$ in a 20 mL vial. 2 mL of different concentrations of HCl (0.1 M, 0.5 M, and 1 M) or pure water was then added and suspended above the $CDCl_3$ solution. The resulting mixture was kept at 20

°C while stirring at a rate of 400 rpm min^{-1}. After different reaction time intervals, 0.5 mL aliquots of the $CDCl_3$ solution phase were taken out with a syringe and transferred to an NMR tube. ^1H NMR spectra of these solutions were measured (Figures S10c-f) and the data were used to establish the reaction kinetics. The proton assignment before and after the dissociation is shown in Figure S10(b).

The extent of conversion in H_2O (i.e., 0 M HCl) or specific concentration of HCl is estimated by the fraction of **aPTHF$_{2k}$** (calculated by integration of the ^1H NMR signals in Figures S10c-f) and plotted in Figure 1(e).

The influence of temperature on the dissociation of **aPTHF$_{2k}$-Btl** was investigated according the following procedures (Figure S11a). Specifically, 200 mg of **aPTHF$_{2k}$-Btl** was dissolved in 4 mL of $CDCl_3$ in a 20 mL vial. 2 mL of 1M HCl was then added and suspended above the $CDCl_3$ solution. The resulting mixture was kept at room temperature (20 °C) or heated to 40 °C, 60 °C while stirring at a rate of 400 rpm min^{-1}. After different reaction time intervals, 0.5 mL aliquots of the $CDCl_3$ solution phase were taken out with a syringe and transferred to an NMR tube. ^1H NMR spectra of these solutions were measured (Figures S11c-e) and the data were used to establish the reaction kinetics. The proton assignment before and after the dissociation is shown in Figure S11(b).

The extent of conversion at specific temperature is estimated by the fraction of **aPTHF$_{2k}$** (calculated by integration of the ^1H NMR signals in Figures S11c-e) and plotted in Figure 1(f).

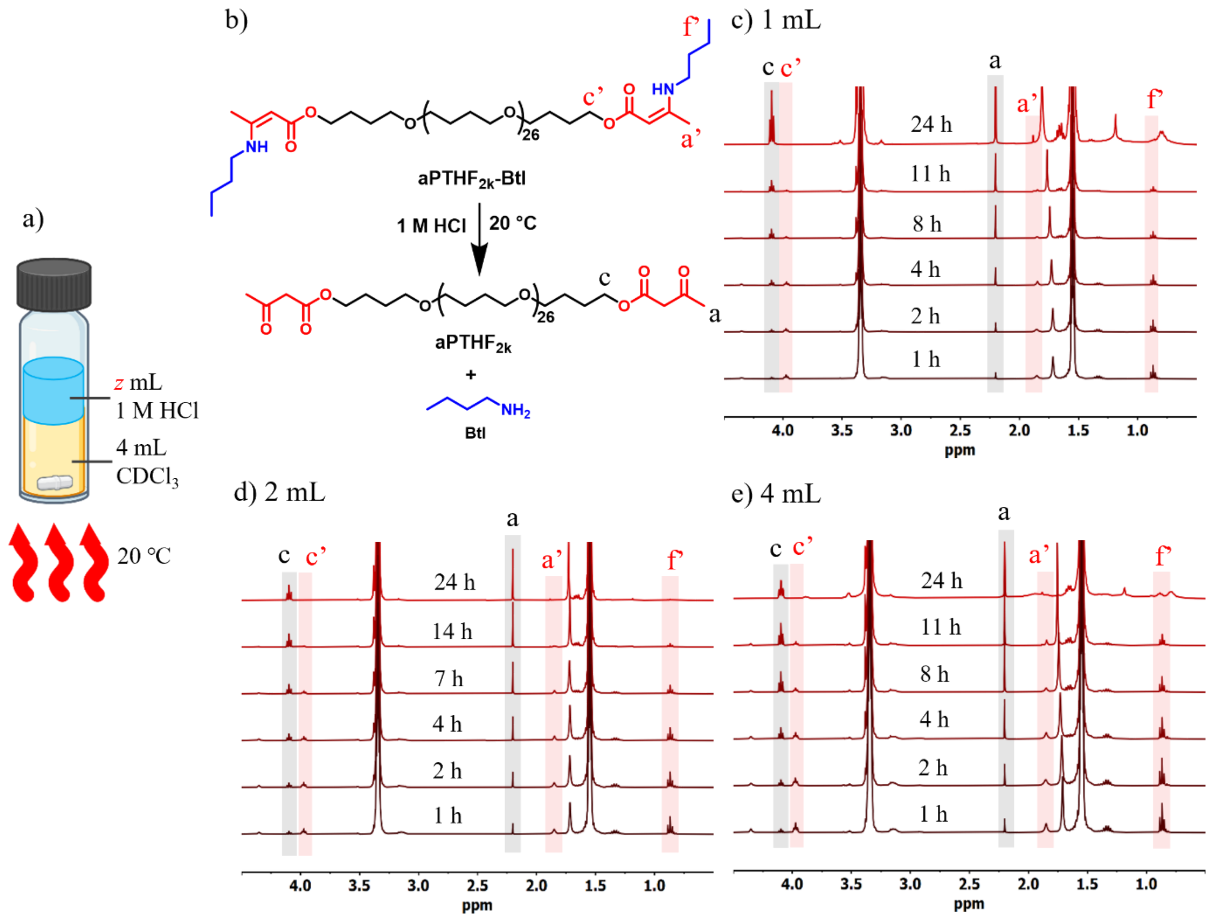

Figure S8. (a) Schematic illustration showing a two-phase mixture solution comprising 1 M HCl (upper phase) and 4 mL of $CDCl_3$ (bottom phase) at 20 °C. (b) Scheme showing the dissociation of **aPTHF$_{2k}$-Btl** into **aPTHF$_{2k}$** and **Btl**, with the assignment of the key protons shown in the ^{1}H NMR spectra. Evolution of the ^{1}H NMR signals ($CDCl_3$, 400 MHz) of the dissociation reaction of **aPTHF$_{2k}$-Btl** (200 mg) in the 1M HCl–$CDCl_3$ mixture solution with the composition illustrated in (a). The samples were collected at different reaction times (as indicated), and the amount of HCl being used was (c) 1 mL, (d) 2 mL, and (e) 4 mL.

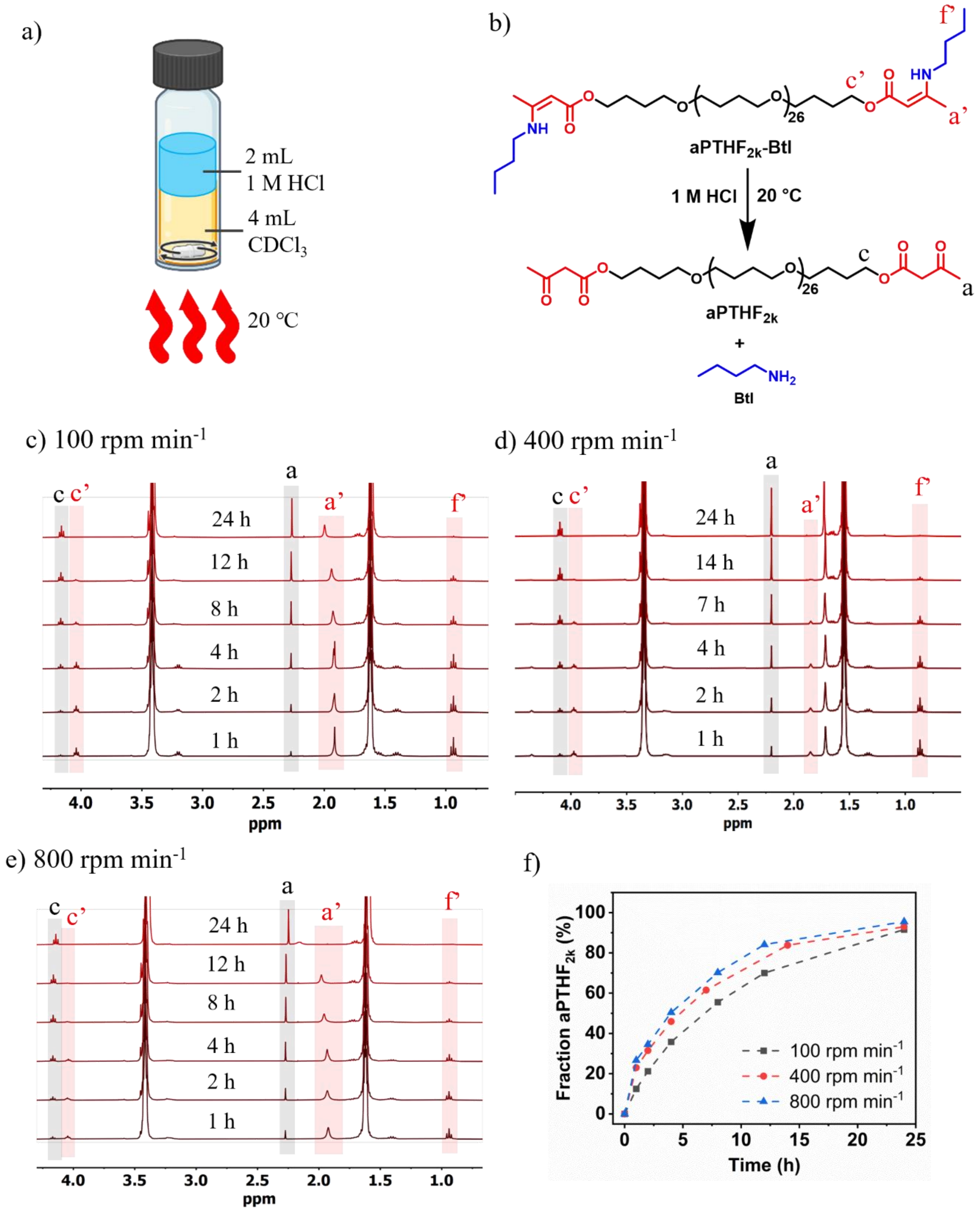

Figure S9. (a) Schematic illustration showing a two-phase mixture solution comprising 2 mL of 1M HCl (upper phase) and 4 mL of $CDCl_3$ (bottom phase) at 20 °C. (b) Scheme showing the dissociation of **aPTHF$_{2k}$-Btl** into **aPTHF$_{2k}$** and **Btl**, with the assignment of the key protons shown in the ^{1}H NMR spectra. Evolution of the ^{1}H NMR signals ($CDCl_3$, 400 MHz) of the dissociation reaction of **aPTHF$_{2k}$-Btl** (200 mg) in the 1M HCl–$CDCl_3$ mixture solution with a stirring rate of (c) 100, (d) 400, or (e) 800 rpm min^{-1}. (f) Extent of conversion of **aPTHF$_{2k}$-Btl** into **aPTHF$_{2k}$** and **Btl**, measured as a function of time at different stirring rates.

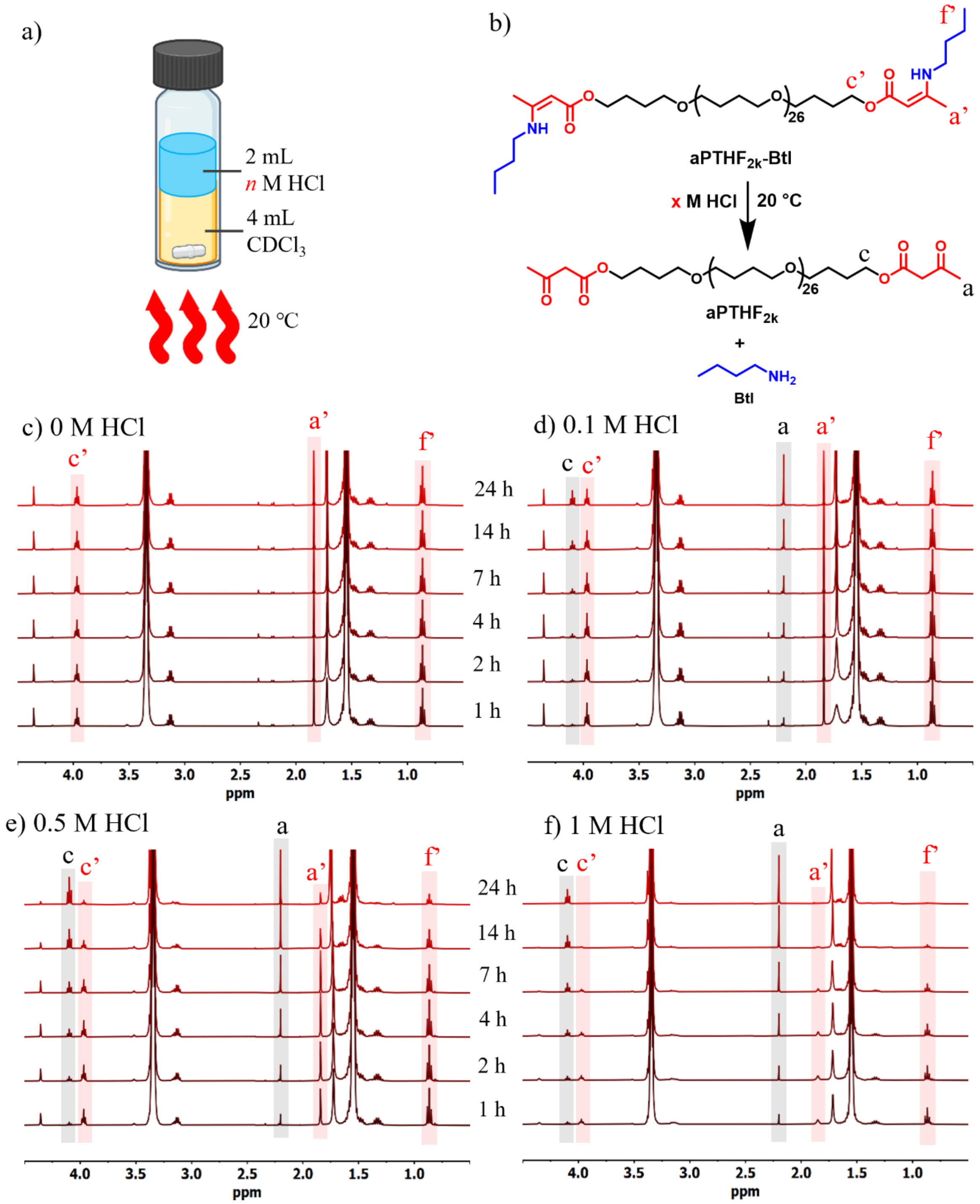

Figure S10. (a) Schematic illustration showing a two-phase mixture solution comprising 2 mL of HCl (upper phase) and 4 mL of $CDCl_3$ (bottom phase) at 20 °C. (b) Scheme showing the dissociation of **aPTHF$_{2k}$-Btl** into **aPTHF$_{2k}$** and **Btl**, with the assignment of the key protons shown in the ^{1}H NMR spectra. Evolution of the ^{1}H NMR signals ($CDCl_3$, 400 MHz) of the dissociation reaction of **aPTHF$_{2k}$-Btl** (200 mg) in the 1M HCl–$CDCl_3$ mixture solution with the composition illustrated in (a). The samples were collected at different reaction times (as indicated), and the concentration of HCl was (c) 0 M, (d) 0.1 M, (e) 0.5 M, and (f) 1 M.

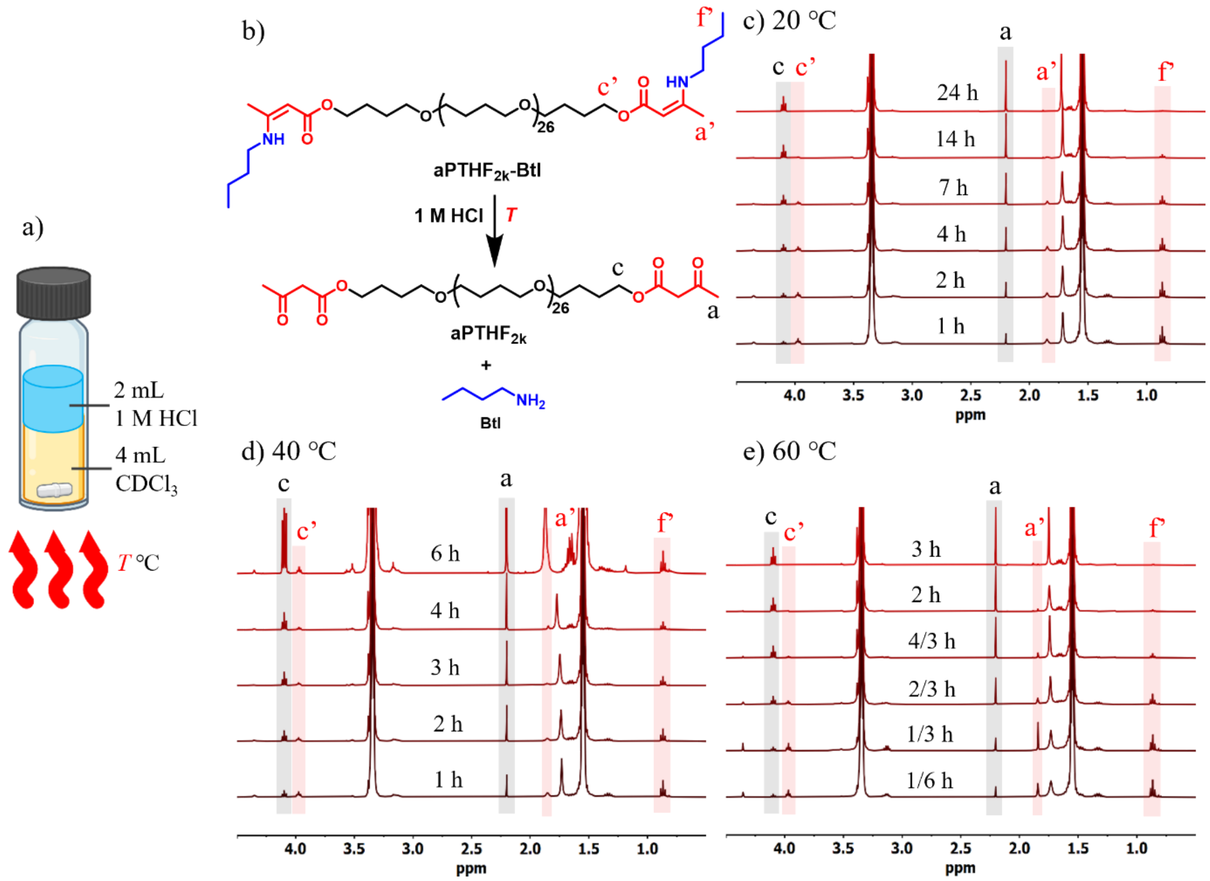

Figure S11. (a) Schematic illustration showing a two-phase mixture solution comprising 2 mL of 1M HCl (upper phase) and 4 mL of $CDCl_3$ (bottom phase) upon heating at different temperatures. (b) Scheme showing the dissociation of **aPTHF$_{2k}$-Btl** into **aPTHF$_{2k}$** and **Btl**, with the assignment of the key protons shown in the ^{1}H NMR spectra. Evolution of the ^{1}H NMR signals ($CDCl_3$, 400 MHz) of the dissociation reaction of **aPTHF$_{2k}$-Btl** (200 mg) in the 1M HCl–$CDCl_3$ mixture solution with the composition illustrated in (a). The samples were collected at different reaction times (as indicated), and the reactions were carried out at (c) 20 °C, (d) 40 °C, and (e) 60 °C.

Determination of the activation energy for the dissociation of aPTHF$_{2k}$-Btl with 1M HCl

The rate constants (k) for the dissociation of **aPTHF$_{2k}$-Btl** with 1M HCl at different temperatures (20, 40, and 60 °C) were obtained by monitoring the fraction of **aPTHF$_{2k}$-Btl** in the reaction mixtures under pseudo first-order condition (large excess of H_2O) (Supplementary Equation 1). The activation energy E_a for the dissociation reaction was determined by plotting ln(k) *vs.* T^{-1} (Supplementary Equation 2).

Supplementary Equation 1:

ln(**aPTHF$_{2k}$-Btl**) = ln(1-fraction **aPTHF$_{2k}$**) = -k(t), the fitting curves are shown in Figure S12(b).

Supplementary Equation 2:

$\ln(k) = \ln(A) - E_a/RT$, the fitting curve is shown in in Figure S12(c).

where T is the temperature expressed in Kelvin, and R = 8.31 J K^{-1} mol^{-1}. The values for k and E_a thus determined are shown in Table S1.

Table S1. Pseudo-first-order rate constants (k) and activation energy (E_a) for the dissociation of **aPTHF$_{2k}$-Btl**.

**Temperature (ºC)**	**Rate constants *k* (s^{-1})**	**Activation energy E_a (kJ mol^{-1})**
20	2.95E-05	57
40	8.85E-05	
60	3.36E-04	

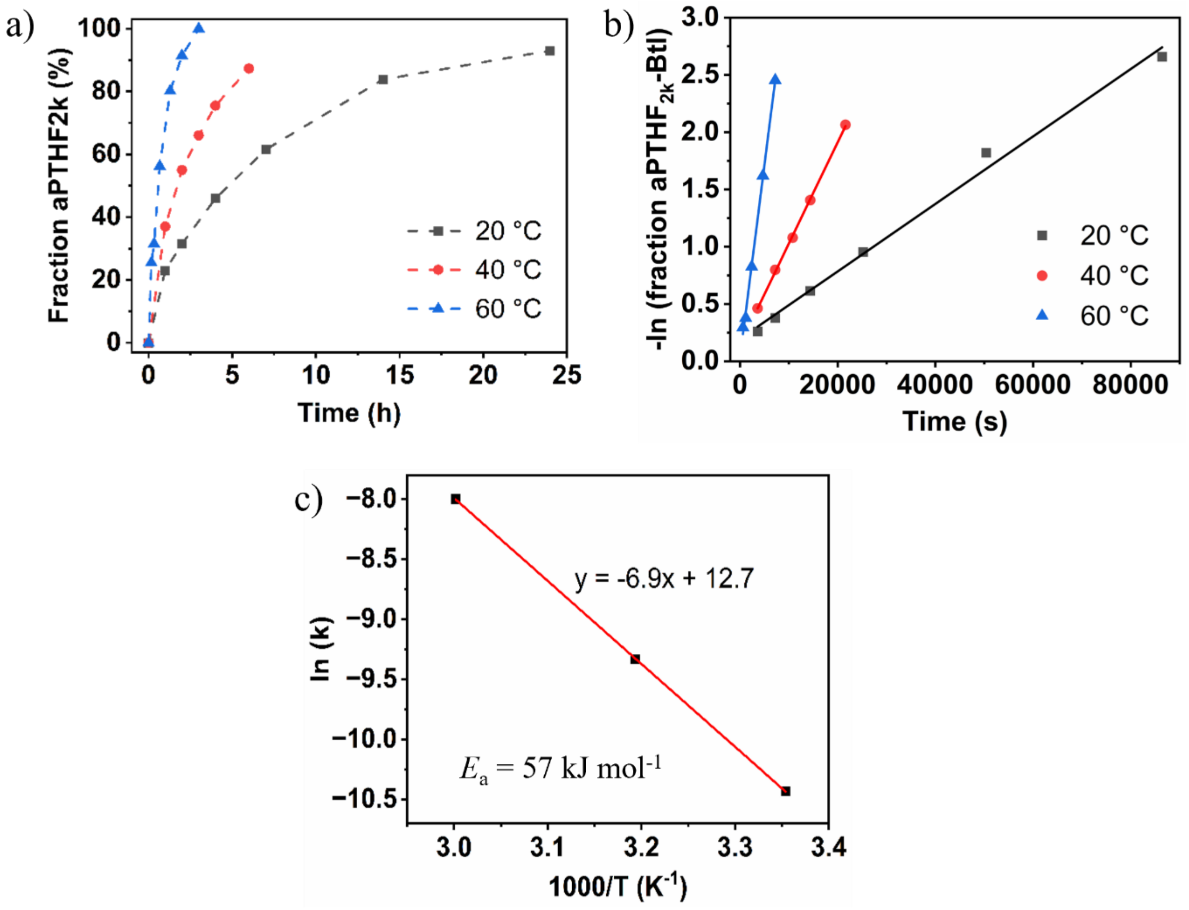

Figure S12. (a) Extent of conversion of **aPTHF$_{2k}$-Btl** into **aPTHF$_{2k}$** and **Btl**, measured as a function of time at 20 ºC (black), 40 ºC (red), 60 ºC (blue) in the presence of a two-phase mixture solution comprising 2 mL of 1M HCl (upper phase) and 4 mL of $CDCl_3$ (bottom phase). The molar fraction of **aPTHF$_{2k}$** was determined by integration of the ^{1}H NMR signals shown in Figure S11. The dashed lines are a simple guide to the eye. (b) Fitting curves for the conversion of **aPTHF$_{2k}$-Btl** into **aPTHF$_{2k}$** and **Btl** shown in (a) at 20 ºC (black), 40 ºC (red), and 60 ºC (blue). (c) Arrhenius plot for the conversion of **aPTHF$_{2k}$-Btl** into **aPTHF$_{2k}$** and **Btl** shown in (a); the analysis affords an activation energy (E_a) of 57 kJ mol^{-1}.

Synthesis of vinylogous urethane polymer networks

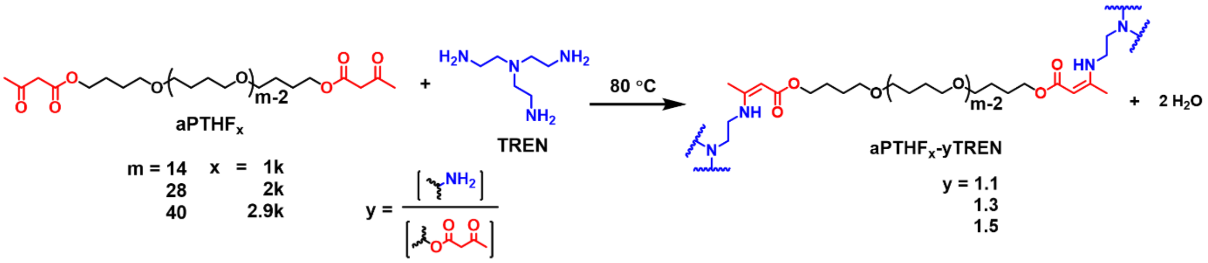

Figure S13. Synthesis of **aPTHF$_x$-yTREN**.

In a typical procedure to synthesize the **VU** polymer, **aPTHF$_x$** (1.2 g for **aPTHF$_{1k}$**, 2.2 g for **aPTHF$_{2k}$**, 3.1 g for **aPTHF$_{2.9k}$**) was dissolved in 10 mL of ethanol in a 20 mL vial. Then, tris(2-aminoethyl)amine (**TREN**) was added to the mixture (reaction scheme shown in Figure S13). The resulting solution was stirred for 10 min at room temperature, and then poured into a Teflon mold. The mixture was heated at 60 °C overnight in a drying oven (no vacuum), and then it was dried by applying vacuum at 80 °C for 12 h. A yellowish solid was obtained, which was labeled as **aPTHF$_x$-yTREN**. **VU** polymers from the reaction of **aPTHF$_{2k}$** and different amounts of excess amine (10, 30, and 50 mol%) were prepared by adjusting the molar ratio of **TREN** added. The specific amounts used for the preparation of **aPTHF$_x$-yTREN** library are reported in Table S2.

Table S2. Amount of **aPTHF$_x$** and **TREN** used in the synthesis of **aPTHF$_x$-yTREN**.

Sample name	aPTHF$_x$		TREN	
	Weight (g)	mmol	Weight (g)	mmol
**aPTHF$_{1k}$-1.3TREN**	1.2	1	0.127	0.87
**aPTHF$_{2k}$-1.3TREN**	2.2	1	0.127	0.87
**aPTHF$_{2.9k}$-1.3TREN**	3.1	1	0.127	0.87
**aPTHF$_{2k}$-1.1TREN**	2.2	1	0.107	0.73
**aPTHF$_{2k}$-1.5TREN**	2.2	1	0.146	1

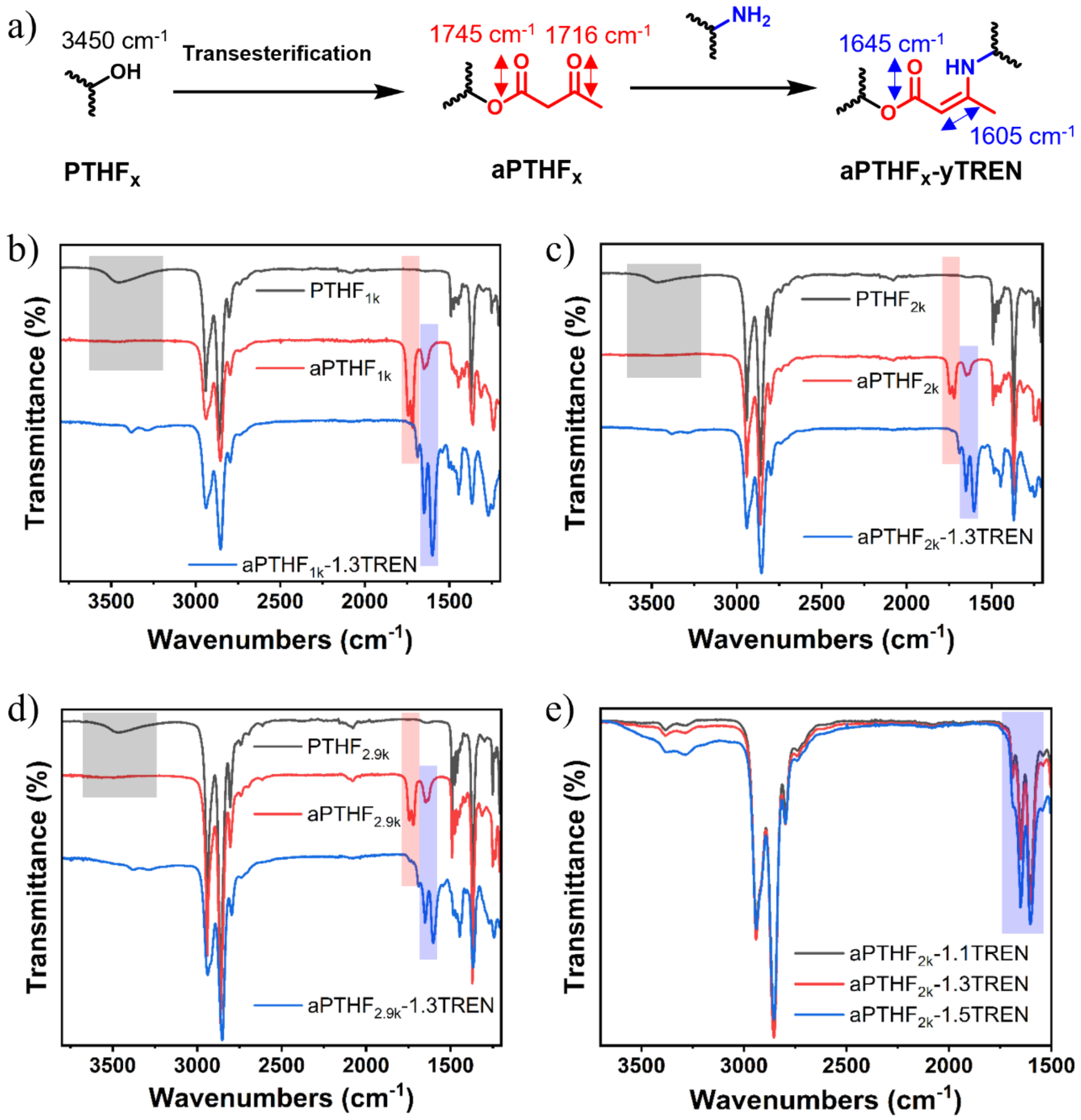

Figure S14. Synthesis of vinylogous urethanes from **PTHF$_x$** (a). FTIR spectra (b-d) showing the conversion of the starting **PTHF$_x$** (black line) into the corresponding **aPTHF$_x$** (red line), and then into **aPTHF$_x$-1.3TREN** (blue line) for **x** = 1k (b), 2k (c), 2.9k (d). FTIR spectra of **aPTHF$_{2k}$-yTREN** with **y** = 1.1 (black line), 1.3 (red line), and 1.5 (blue line) (e). The grey areas highlight the spectrum region of the -*OH* stretching vibrations (around 3450 cm^{-1}). The red areas highlight the spectrum region of carbonyl stretching vibrations (1716 cm^{-1}, 1745 cm^{-1}). The blue areas highlight the spectrum region associated to the stretching vibrations of the carbonyl groups (1645 cm^{-1}) and double bonds (1605 cm^{-1}).

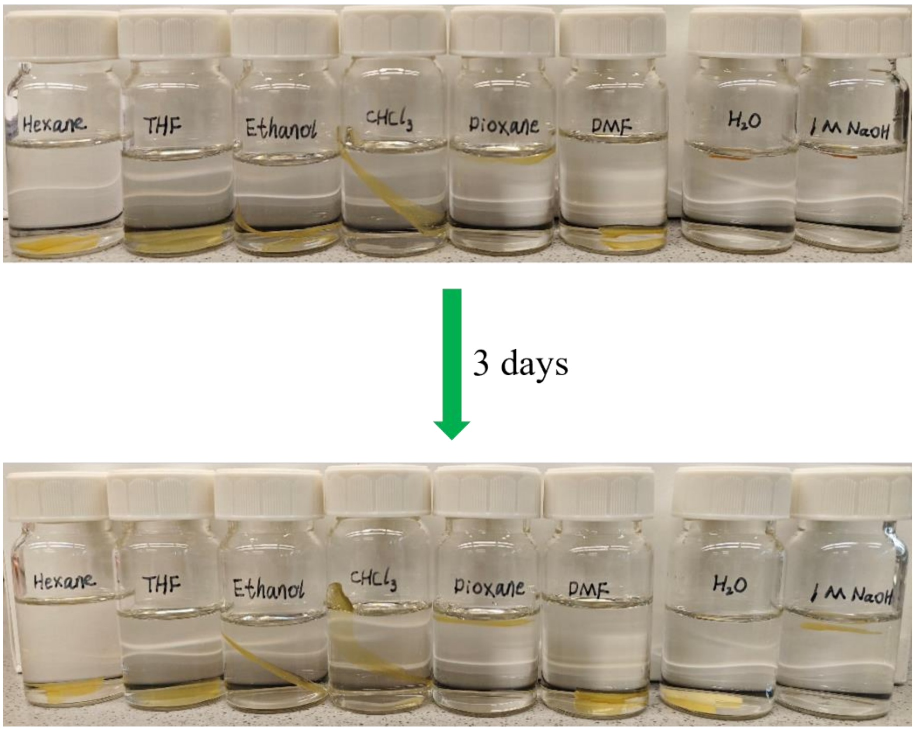

Figure S15. Photographs of **aPTHF$_{2k}$-1.3TREN** films dispersed in 10 mL of various organic solvents including hexane, THF, ethanol, $CHCl_3$, dioxane, DMF, H_2O, and 1M NaOH (as indicated) before (top row) and after (bottom row) a 3-day incubation period at room temperature.

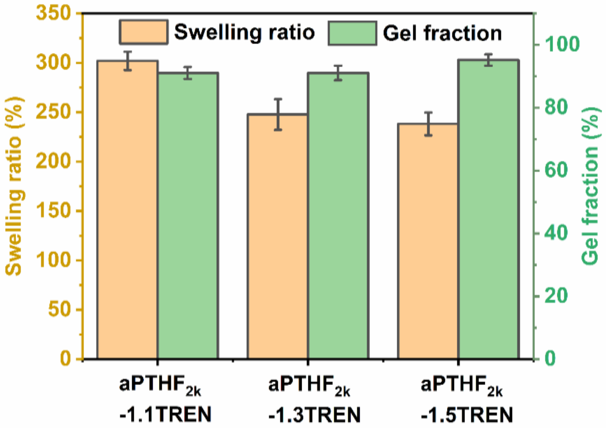

Figure S16. Swelling ratio (yellow) and gel fraction (green) of**aPTHF$_{2k}$-yTREN** (**y** = 1.1, 1.3, and 1.5).

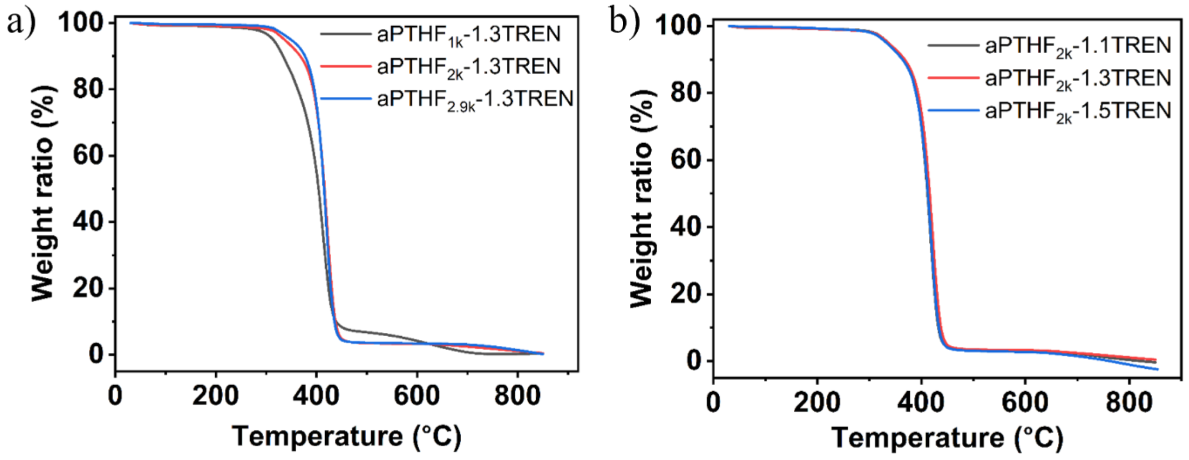

Figure S17. TGA curves of (a) **aPTHF$_x$-1.3TREN** with **x** = 1k (black), 2k (red), and 2.9k (blue), and (b) **aPTHF$_{2k}$-yTREN** with **y** = 1.1 (black), 1.3 (red), and 1.5 (blue).

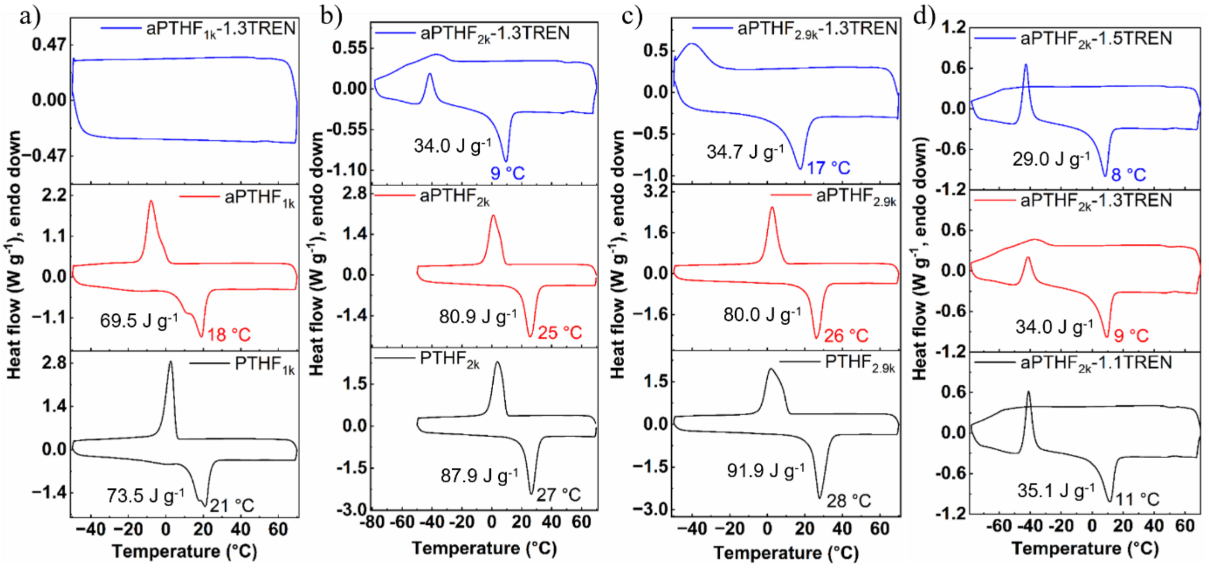

Figure S18. DSC traces of the second heating/cooling runs for **PTHF$_x$** (black line), **aPTHF$_x$** (red line), **aPTHF$_x$-1.3TREN** (blue line) with **x** = 1k (a), 2k (b), and 2.9k (c), and **aPTHF$_{2k}$-yTREN** with **y** = 1.1 (black), 1.3 (red), and 1.5 (blue) (d). Fusion enthalpy ΔH_c is calculated by integration of the melting peak.

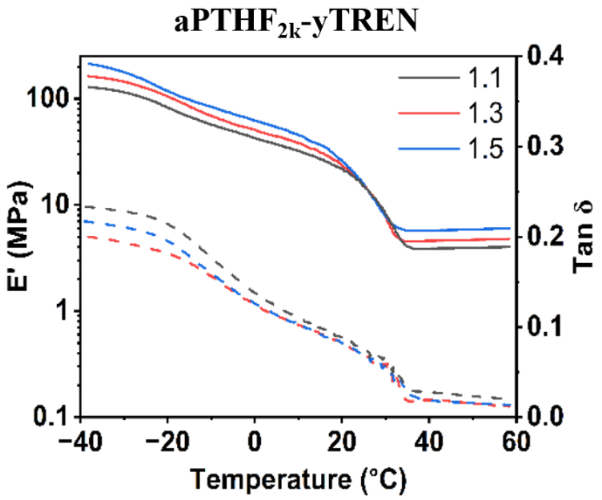

Figure S19. DMA traces showing the storage modulus (*E′*) (solid lines) and tan δ (dashed lines) of the **aPTHF$_{2k}$-yTREN** series (**y** = 1.1, 1.3, and 1.5) as a function of temperature.

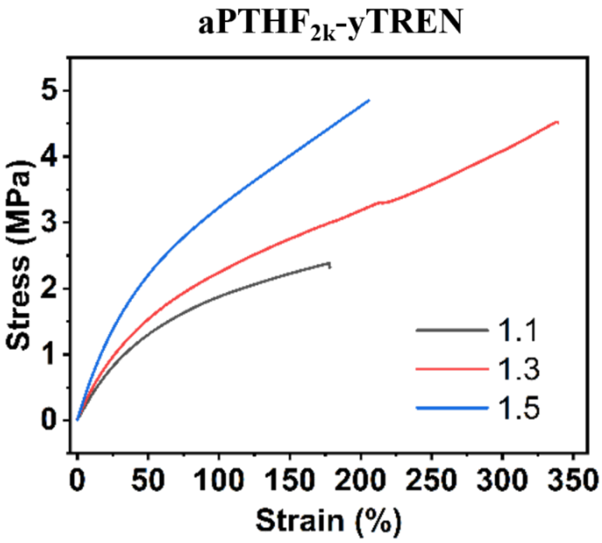

Figure S20. Stress-strain curves of the **aPTHF$_{2k}$-yTREN** series (**y** = 1.1, 1.3, and 1.5).

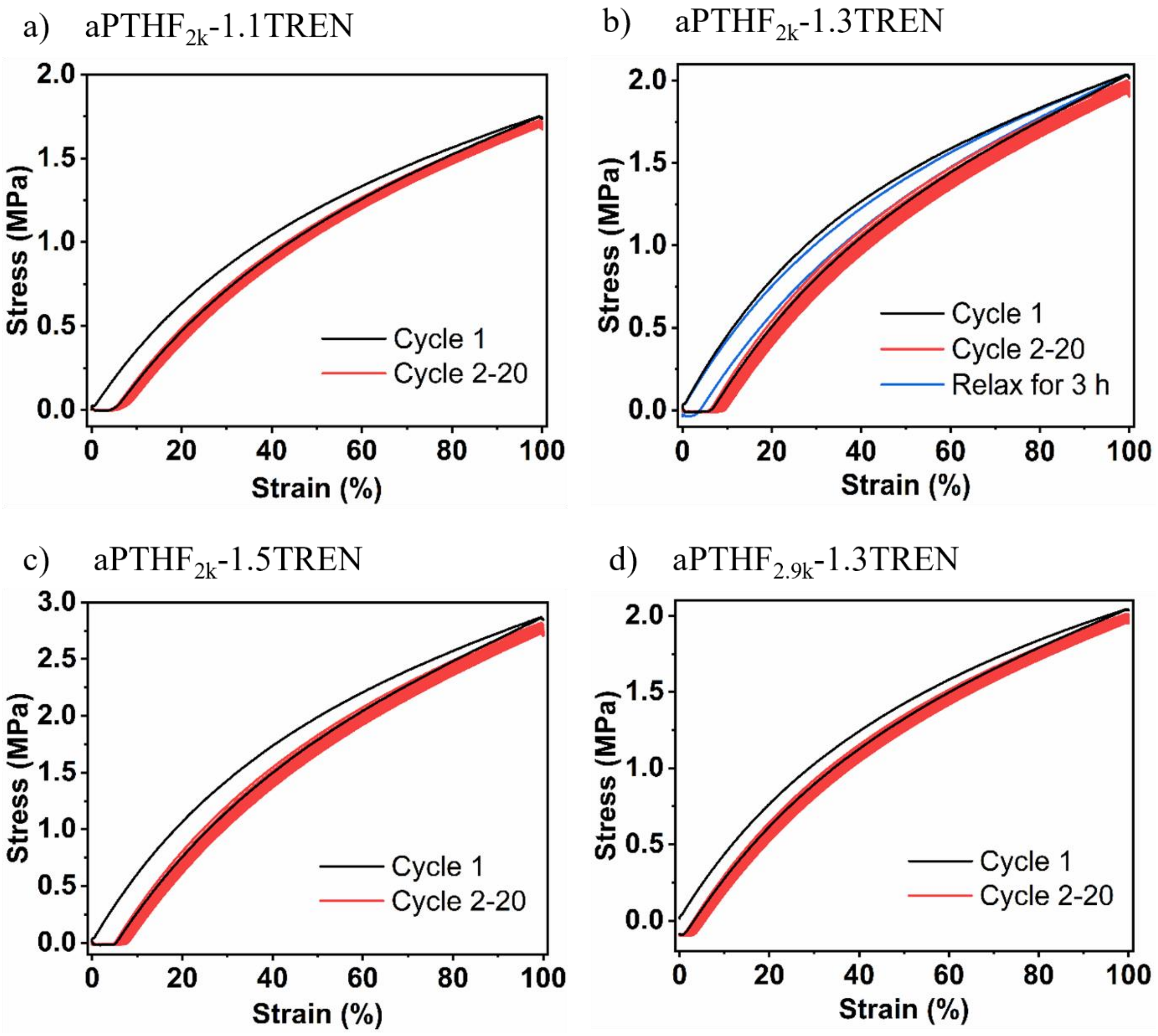

Figure S21. Cyclic tensile curves of (a) **aPTHF$_{2k}$-1.1TREN**, (b) **aPTHF$_{2k}$-1.3TREN**, (c) **aPTHF$_{2k}$-1.5TREN**, and (d) **aPTHF$_{2.9k}$-1.3TREN**, measured at a maximum loading strain of 100% for consecutive 20 cycles with the first cycle highlighted in black and the rest in red. For **aPTHF$_{2k}$-1.3TREN**, one more cyclic tensile test was made with a 3-hour relaxing time under room temperature after the consecutive 20 cycles.

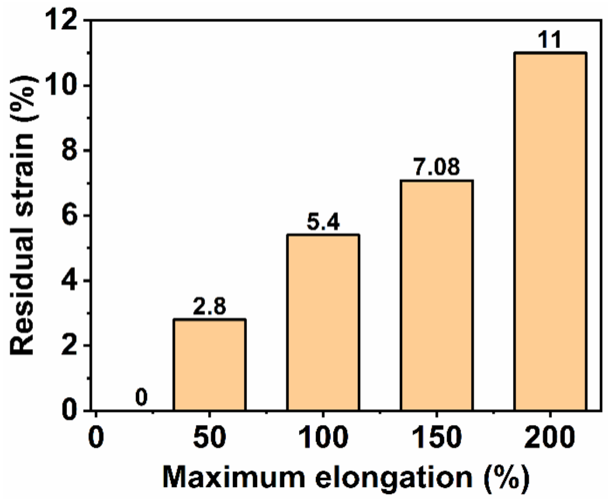

Figure S22. The residual strains of **aPTHF$_{2.9k}$-1.3TREN** measured at the increased maximum loading strains from 20% to 200%.

Activation energy for aPTHF$_x$-yTREN networks

The activation energy was determined relying on the Maxwell model for stress-relaxation,[1] according to Supplementary Equation (3):

$\sigma_t/\sigma_0 = e^{-t/\tau}$,

where σ_t and σ_0 are the stress at time t and the initial stress, respectively, and τ is the relaxation time. By convention, the relaxation time is taken as the time at which the normalized stress is equal to 1/e (ca. 0.37).

The obtained relaxation times (τ) measured at different temperatures (Figures S23-24, Table S3) were fit to Supplementary Equation (4):

$\ln\tau(T) = \ln\tau_0 + E_a/R\mathrm{T}$

where R is the universal gas constant (8.31 J K^{-1} mol^{-1}), and T is the temperature expressed in Kelvin degrees. $\tau(T)$ is relaxation time obtained at temperature T.

The Arrhenius fits are shown in Figures S23(d), S24(d) and the calculated activation energies are shown in Table S3.

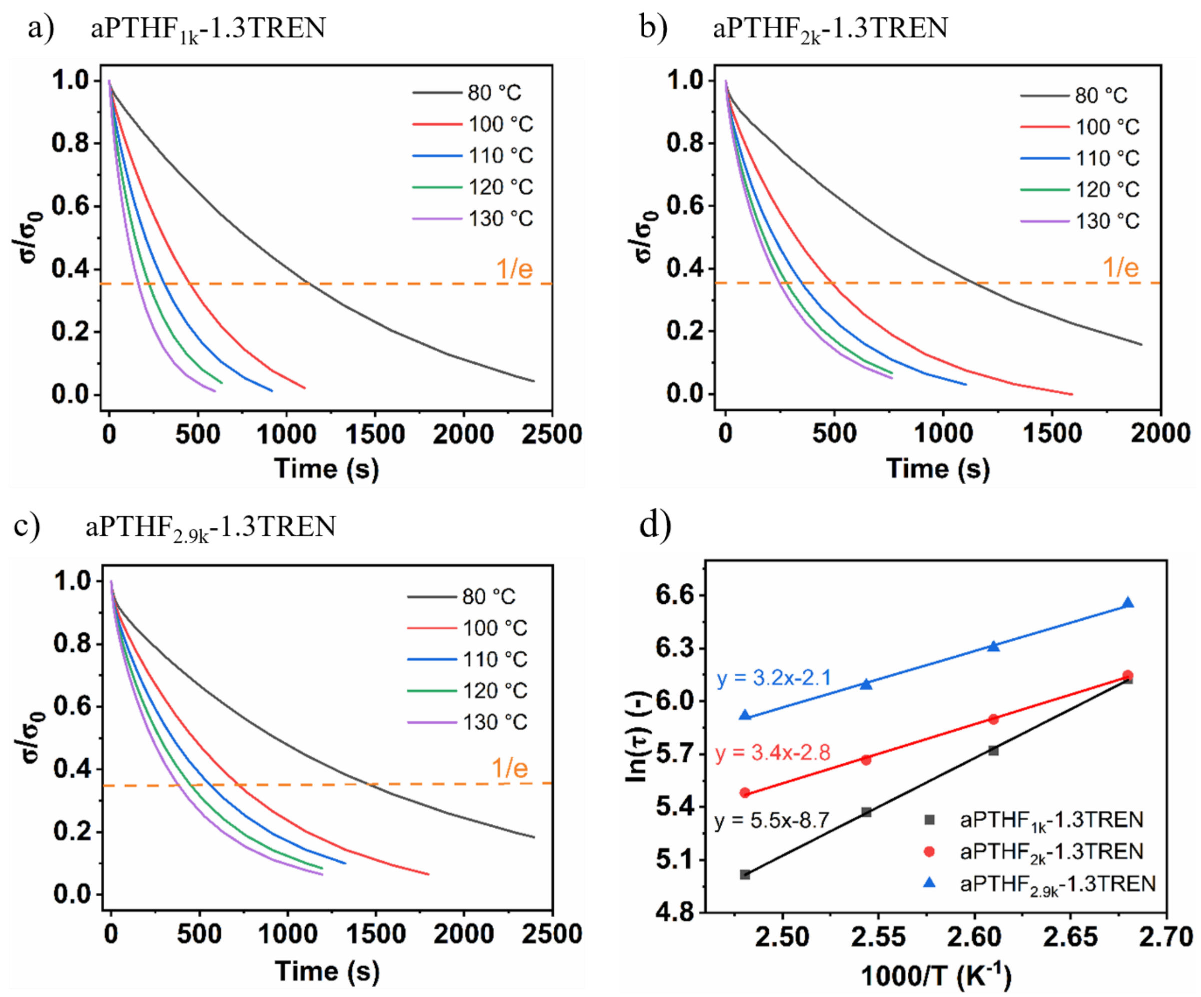

Figure S23. Stress relaxation curves of **aPTHF$_x$-1.3TREN** with **x** = 1k (a), 2k (b), and 2.9k (c) as a function of time at 80 °C (black), 100 °C (red), 110 °C (blue), 120 °C (green), 130 °C (violet), and (d) the corresponding Arrhenius plots for the stress-relaxation.

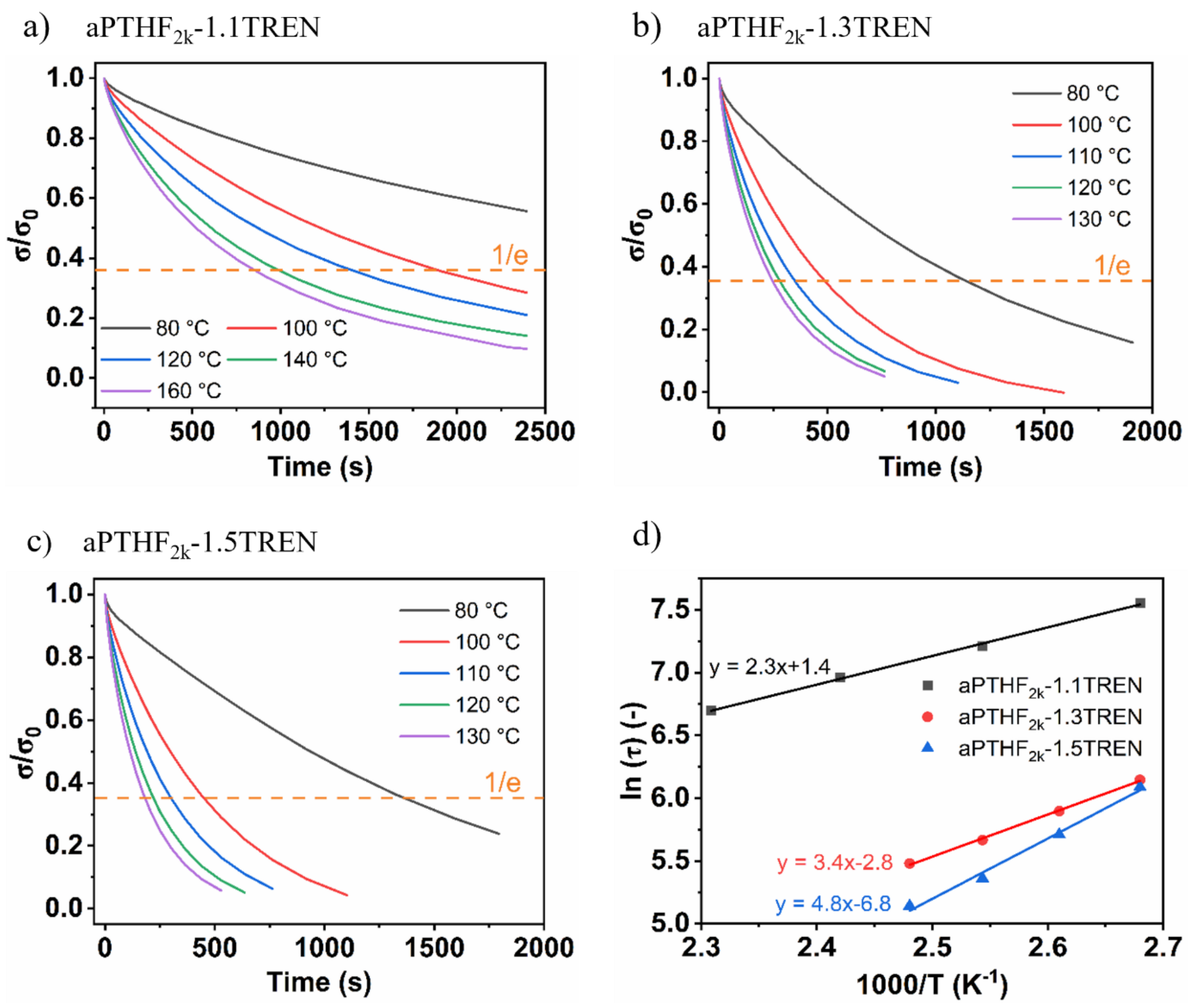

Figure S24. Stress relaxation curves of **aPTHF$_{2k}$-yTREN** with **y** = 1.1 (a), 1.3 (b), and 1.5 (c) as a function of time at different temperatures (as indicated), and (d) the corresponding Arrhenius plots for the stress-relaxation.

Table S3. Summary of stress relaxation times $\tau(T)$ at different temperatures and activation energies E_a.

Sample name	Temperature (°C)	relaxation time (s)	Arrhenius fitting equations; Activation energies (kJ mol^{-1})
**aPTHF$_{1k}$-1.3TREN**	80	1151	y = 5.5x-8.7, R^2 = 99.9%; 46
	100	458	
	110	305	
	120	215	
	130	151	
**aPTHF$_{2k}$-1.3TREN**	80	1101	y = 3.4x-2.8, R^2 = 99.8%; 28
	100	467	
	110	364	
	120	289	
	130	240	
**aPTHF$_{2.9k}$-1.3TREN**	80	1456	y = 3.2x-2.1, R^2 = 99.7% 26
	100	702	
	110	547	
	120	441	
	130	371	
**aPTHF$_{2k}$-1.1TREN**	80	–	y = 2.3x+1.4, R^2 = 99.8%; 19
	100	1908	
	120	1353	
	140	1056	
	160	810	
**aPTHF$_{2k}$-1.5TREN**	80	1310	y = 4.8x-6.8, R^2 = 99.6; 40
	100	441	
	110	302	
	120	213	
	130	171	

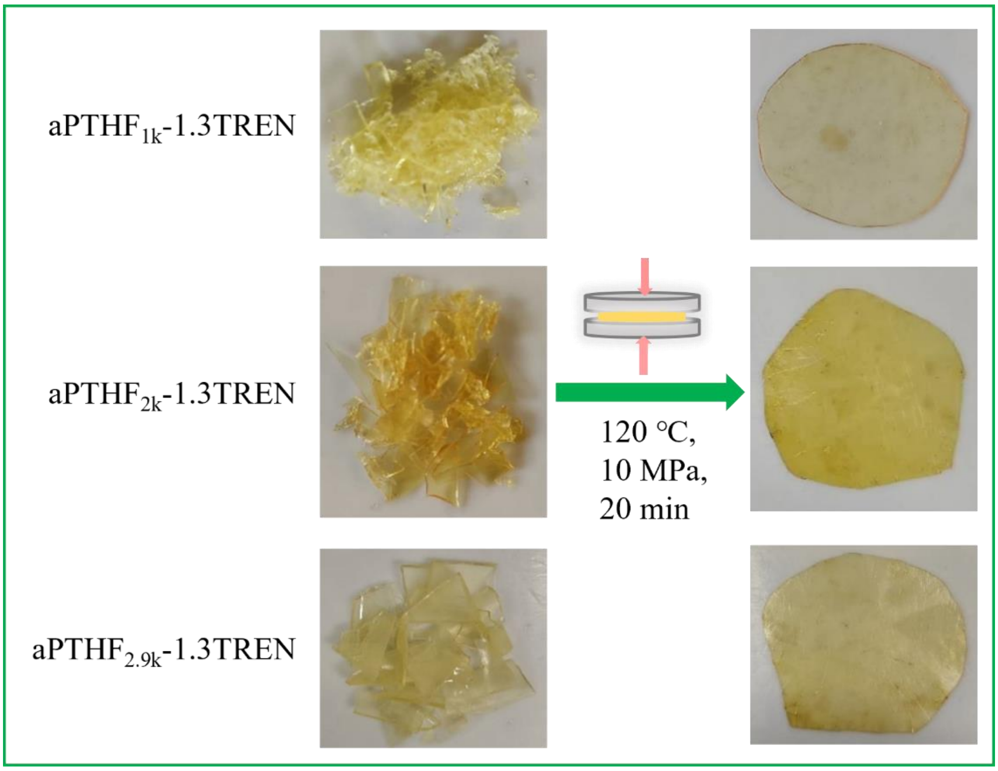

Figure S25. Thermal reprocessing of **aPTHF$_x$-1.3TREN** with **x** = 1k, 2k, and 2.9k from small pieces of materials (left) into new films (right) by compression-molding (T = 120 °C; P = 10 MPa; time = 20 min).

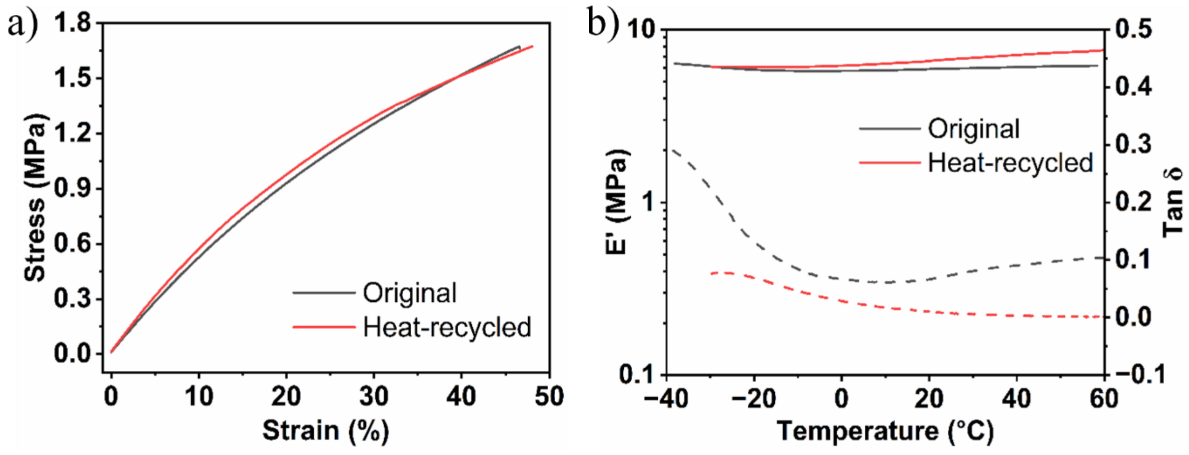

Figure S26. (a) Stress-strain curves and (b) DMA traces of the original **aPTHF$_{1k}$-1.3TREN** film, and recycled film produced by compression-molding at 120 °C.

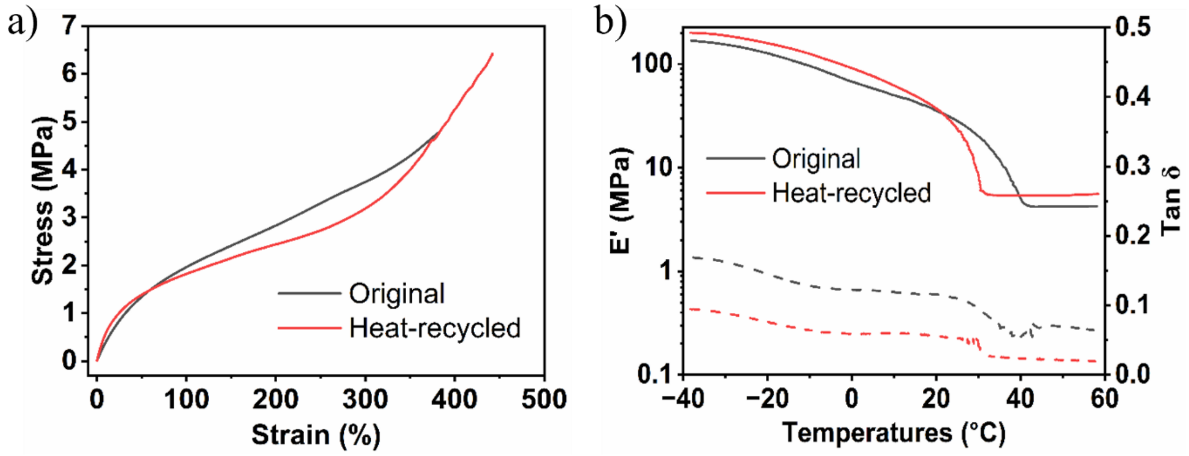

Figure S27. (a) Stress-strain curves and (b) DMA traces of the original **aPTHF$_{2.9k}$-1.3TREN** film, and recycled film produced by compression-molding at 120 °C.

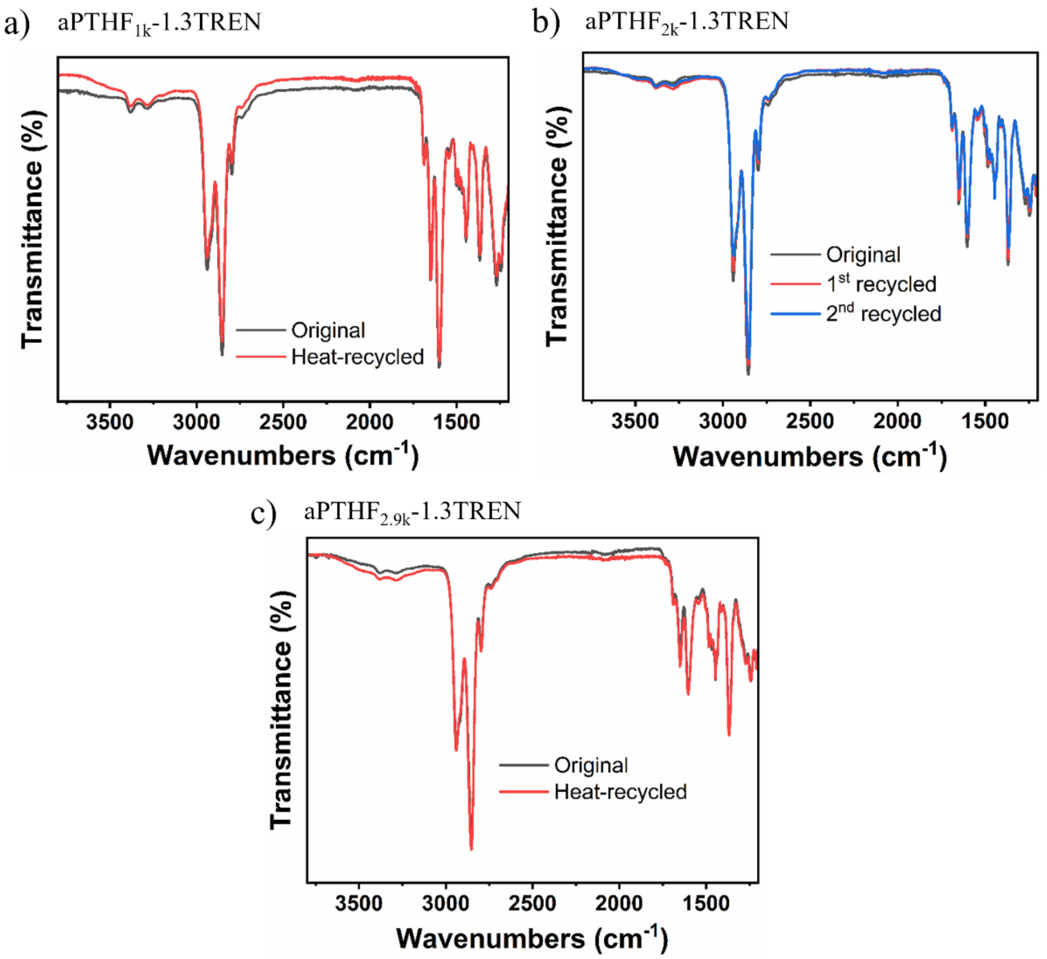

Figure S28. FTIR spectra of **aPTHF$_x$-1.3TREN** with **x** = (a) 1k, (b) 2k, and (c) 2.9k before and after the thermal reprocessing process.

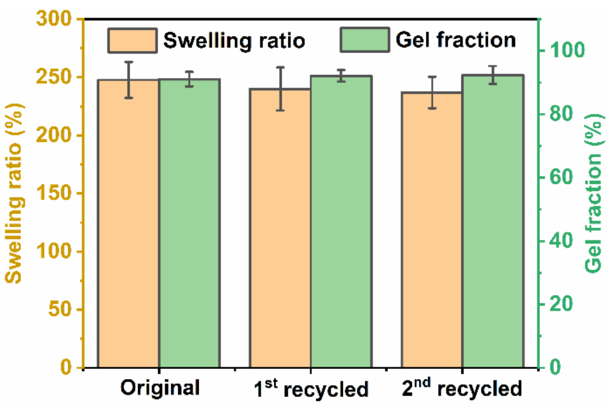

Figure S29. Swelling ratio and gel fraction of **aPTHF$_{2k}$-1.3TREN** before and after the thermal reprocessing process.

Depolymerization of $aPTHF_x$-1.3TREN and subsequent recovery of starting materials

0.5 g of **$aPTHF_x$-1.3TREN** shreds (x = 1k, 2k, 2.9k) was added to 15 mL of 1M HCl in a small vial while stirring at a rate of 400 rpm min^{-1}. **$aPTHF_{1k}$-1.3TREN** got disintegrated and the solution turned cloudy over time. **$aPTHF_{2k}$-1.3TREN** gradually lost shape and stuck to the stir bar in 18 h. While for **$aPTHF_{2.9k}$-1.3TREN**, it maintained a good dimension, albeit with color turning opaque (Figure S30, top). To further depolymerize the samples, 15 mL of diethyl ether was added to the depolymerized mixture and the resulting two-phase mixture solution was stirred at 400 rpm min^{-1} for another 24 h. It resulted in a clear two-phase mixture solution (Figure S30, bottom).

To recover **$aPTHF_x$**, the upper phase ether solution was taken out using a separation funnel. The separated solution was washed with pure water three times and then dried by magnesium sulfate ($MgSO_4$). $MgSO_4$ was filtered out and the diethyl ether was removed by rotary evaporation. After vacuum drying at 80 °C for 24 h, yellowish liquid (**$aPTHF_{1k}$**) or solid (**$aPTHF_{2k}$** and **$aPTHF_{2.9k}$**) materials were obtained.

Recovered **$aPTHF_{1k}$**: yield 90.0%, 0.45 g; ^{1}H NMR (400 MHz, $CDCl_3$) δ 4.12 (t, *J* = 6.6 Hz, 2H), 3.40 (d, *J* = 2.8 Hz, 2H), 3.36 (d, *J* = 5.3 Hz, 25H), 2.22 (s, 3H), 1.57 (p, *J* = 2.9 Hz, 27H).

Recovered **$aPTHF_{2k}$**: yield 98.1%, 0.49 g; ^{1}H NMR (400 MHz, $CDCl_3$) δ 4.17 (t, *J* = 6.6 Hz, 2H), 3.45 (s, 2H), 3.42 (q, *J* = 4.6 Hz, 53H), 2.27 (s, 3H), 1.62 (p, *J* = 3.1 Hz, 54H).

Recovered **$aPTHF_{2.9k}$**: yield 98.5%, 0.49 g; ^{1}H NMR (400 MHz, $CDCl_3$) δ 4.17 (t, *J* = 6.6 Hz, 2H), 3.45 (s, 2H), 3.41 (t, *J* = 5.3 Hz, 78H), 2.27 (s, 3H), 1.62 (p, *J* = 3.1 Hz, 80H).

The comparisons between the ^{1}H NMR spectra of the original and recovered **$aPTHF_x$** are shown in Figures S31-32, 3e.

To recovered **TREN**, for the sake of operation, the bottom 1M HCl solution was collected and combined together from these three vials. The obtained solution was washed by diethyl ether gently three times. Next, ca. 45 mL of 1M NaOH was added to the acid solution until it became slightly alkaline, during which a pH paper serves as the indicator. The resulting solution was dried by rotary evaporation before 10 mL of $CHCl_3$ was added to extract the organic compound from the mixture. Finally, the obtained $CHCl_3$ solution was filtered using a syringe filter, and the $CHCl_3$ was removed by rotary evaporation, leading to the formation of yellowish liquid crude. The **TREN** was recovered by vacuum drying the crude at 60 °C for 24 h.

Recovered **TREN**: yield 86.1 %, 0.081 g; ^{1}H NMR (400 MHz, $CDCl_3$) δ 2.77 (t, *J* = 6.1 Hz, 1H), 2.52 (t, *J* = 6.1 Hz, 1H), 1.38 (s, 1H).

The comparison between the ^{1}H NMR spectra of the original and recovered **TREN** is shown in Figure 3f.

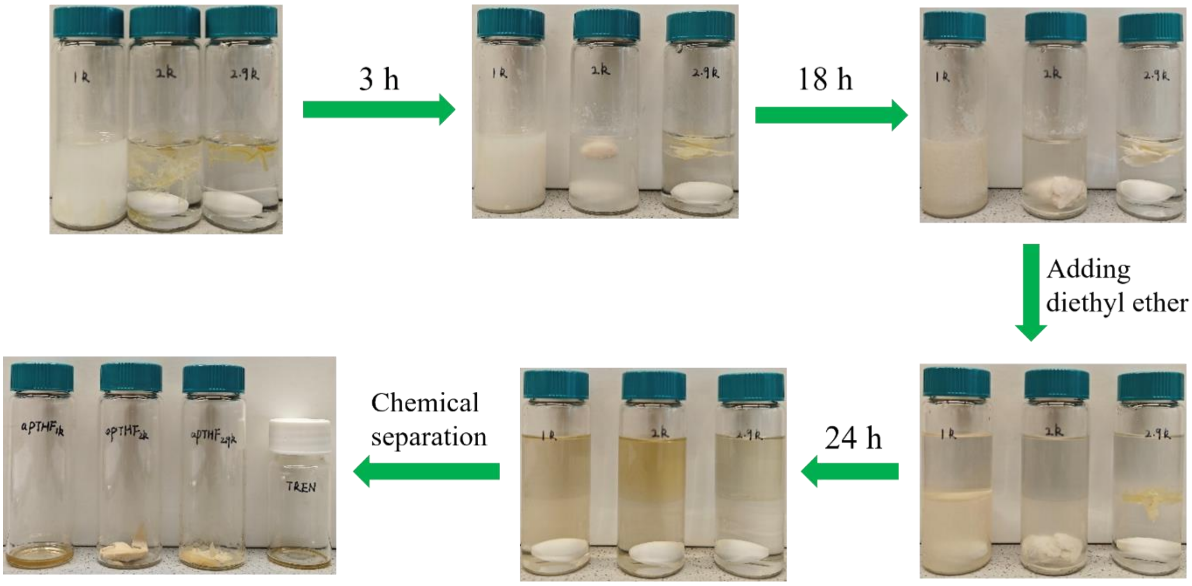

Figure S30. Photographs showing the depolymerization of **aPTHF$_x$-1.3TREN** shreds (**x** = 1k, 2k, 2.9k) by 15 mL of 1M HCl as a function of time (top), followed by the addition of 15 mL of diethyl ether for another 24-hour depolymerization, and the final separation and recovery of **aPTHF$_x$** and **TREN** (bottom).

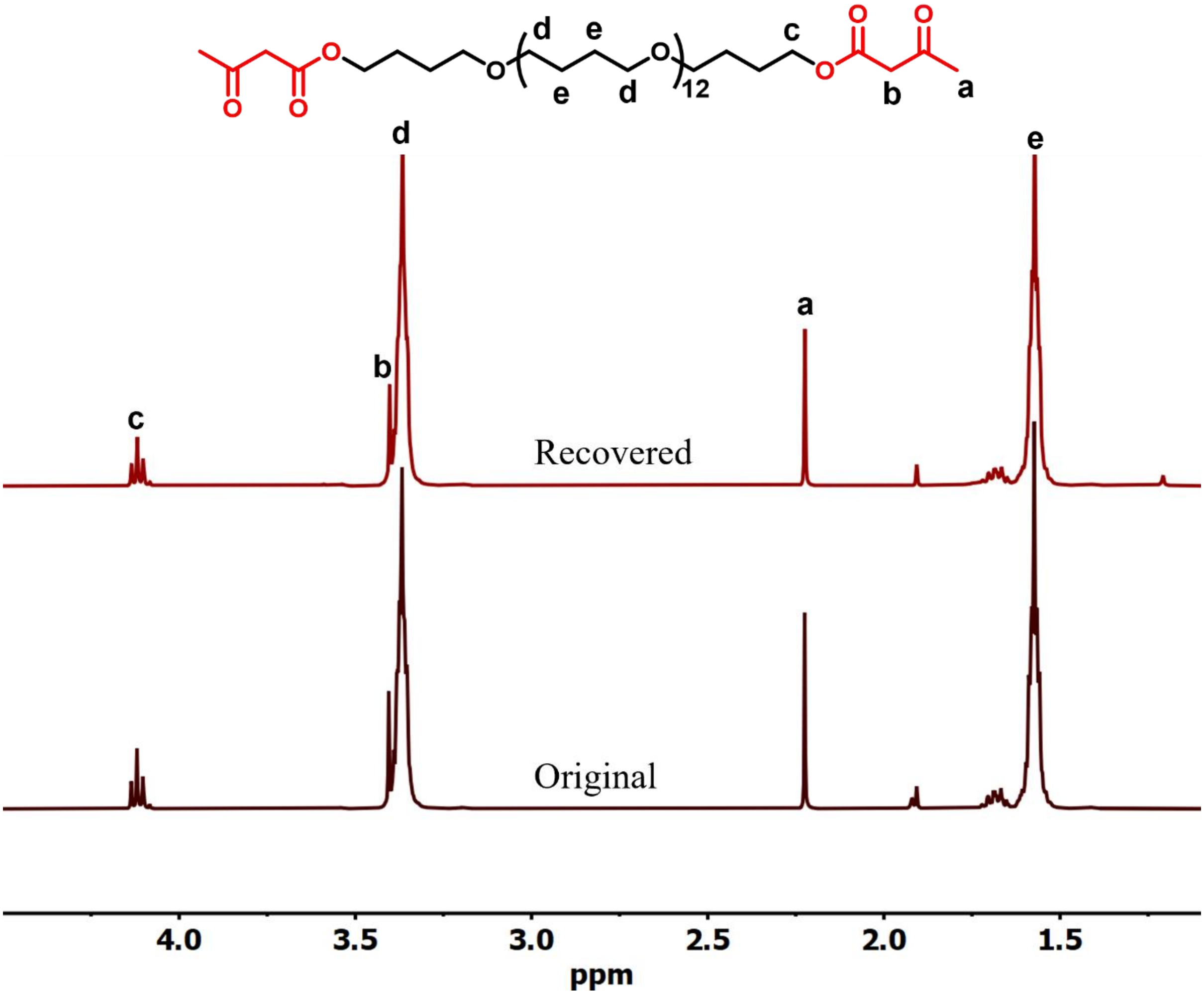

Figure S31. Comparison between the ^{1}H NMR spectra ($CDCl_3$, 400 MHz) of the original and recovered **aPTHF$_{1k}$**.

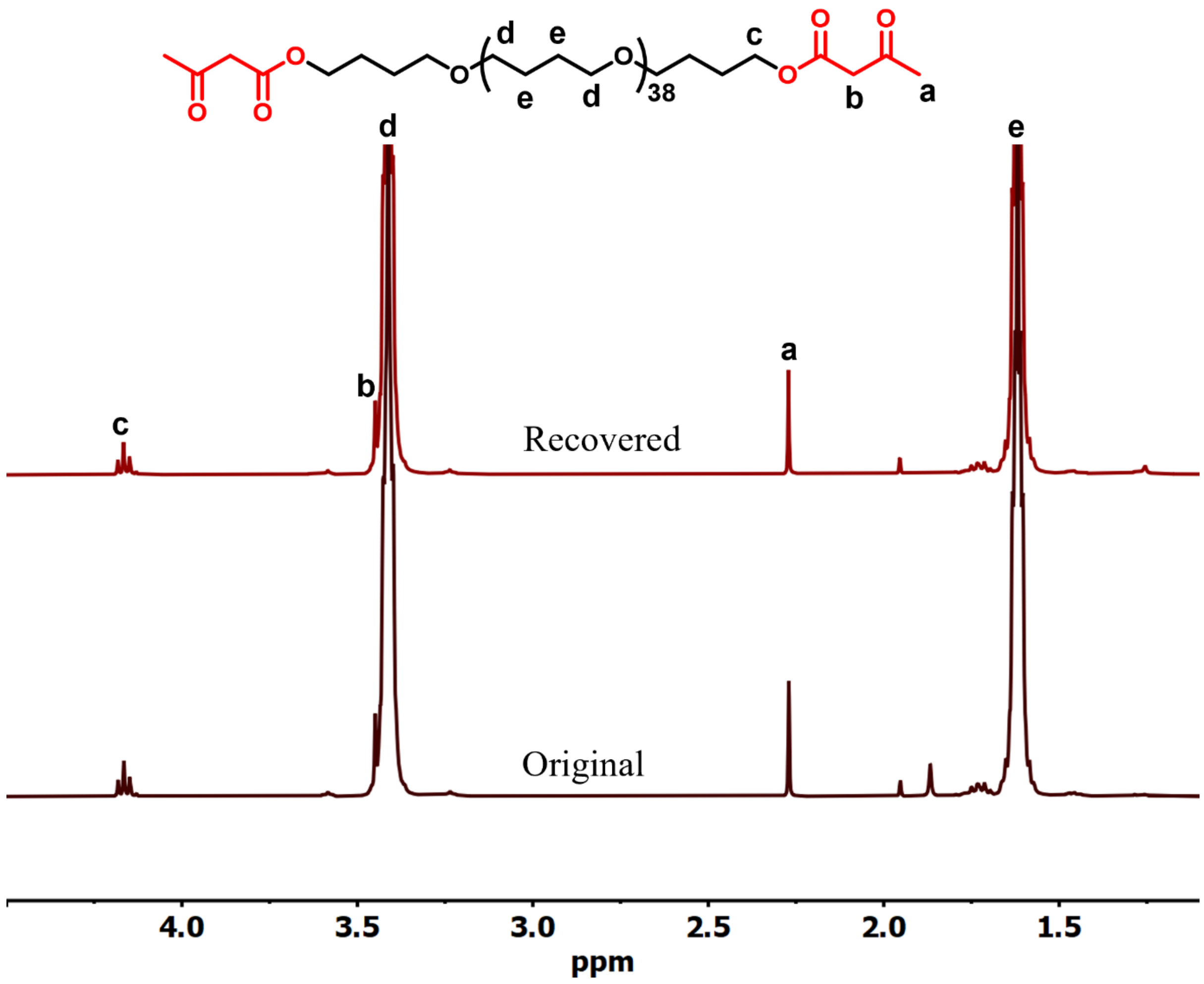

Figure S32. Comparison between the ^{1}H NMR spectra ($CDCl_3$, 400 MHz) of the original and recovered **aPTHF$_{2.9k}$**.

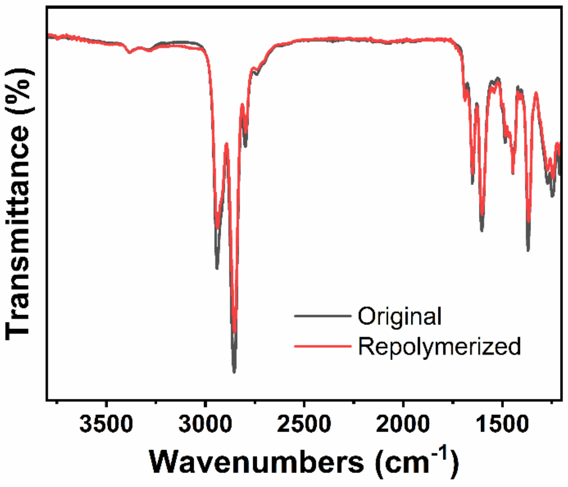

Figure S33. FTIR spectra of the original (black) and repolymerized (red) **aPTHF$_{2k}$-1.3TREN**.

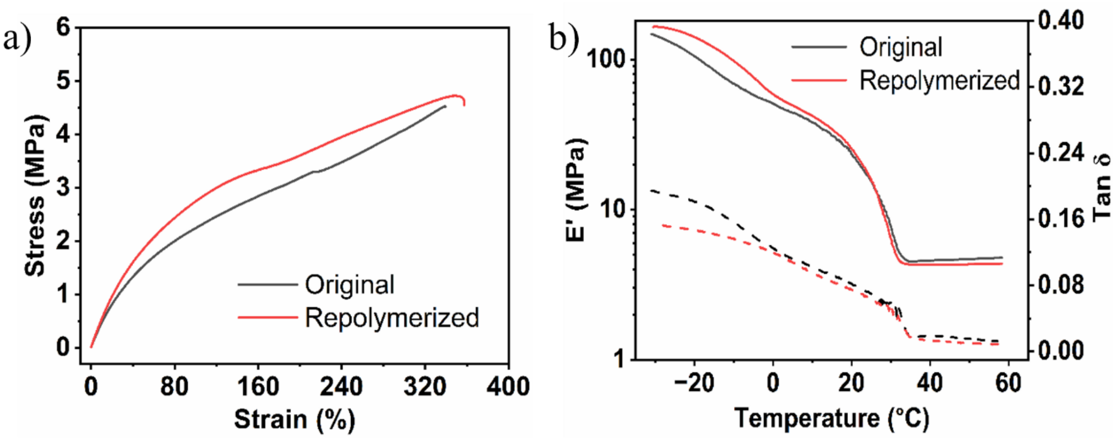

Figure S34. (a) Stress-strain curves and (b) DMA traces of the original (black) and repolymerized (red) **aPTHF$_{2k}$-1.3TREN** films.

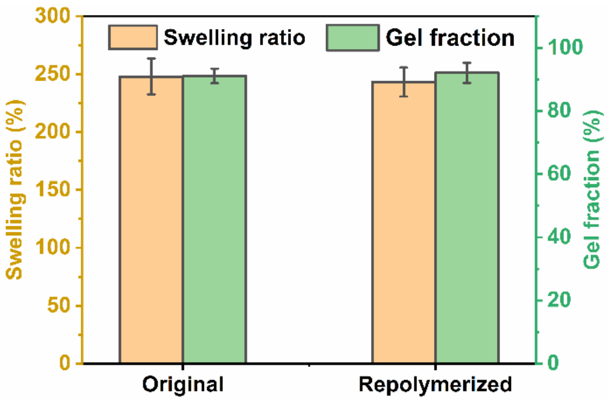

Figure S35. Swelling ratio and gel fraction of the original and repolymerized **aPTHF$_{2k}$-1.3TREN**.

Synthesis, depolymerization, and recovery of starting materials of strain sensor

The synthesis of strain sensor is similar to the synthesis of **aPTHF$_x$-yTREN** polymers. **aPTHF$_{2k}$** (2.2 g, 1 mmol) was dissolved in 10 mL of ethanol in a 20 mL vial. Then, **TREN** (0.127 g, 0.87 mmol) and multi-walled carbon nanotubes (**MCNs**; 0.116 g) were added to the mixture. The resulting solution was stirred for 10 min at room temperature, and then poured into a Teflon mold. The mixture was heated at 60 °C overnight in a drying oven (no vacuum), and then it was dried by applying vacuum at 80 °C for 12 h. A black solid material was obtained, which was labelled as **VU/5%MCNs**. Before being applied as a strain sensor, the **VU/5%MCNs** composite was flatten by a compression-molding process at 120 °C, 10 MPa for 20 min with a distance control of 0.2 mm.

The depolymerization of **VU/5%MCNs** was carried out by treating 0.5 g of **VU/5%MCNs** with a two-phase mixture solution comprising 10 mL of 1M HCl and 10 mL of diethyl ether (visual illustration shown in Figure 5a). The mixture was kept at room temperature for 24 h while stirring at a rate of 400 rpm min^{-1}. After this time, **aPTHF$_{2k}$-1.3TREN** was completely dissolved, and **MCNs** were concentrated in the HCl solution (bottom phase).

The **MCNs** could be recovered by vacuum filtration, and washed by appropriate amounts of diethyl ether before characterization. The morphology and chemical structure of the recovered **MCNs** were investigated by SEM technique equipped with EDX analysis and then compared with those of the original **MCNs** (Figures 5c, S36). The upper diethyl ether solution was then taken out using the separation funnel to recover **aPTHF$_{2k}$**. The separated solution was washed with pure water three times and then dried by $MgSO_4$. The $MgSO_4$ was filtered out and the diethyl ether was removed by rotary evaporation. After vacuum drying at 80 °C for 24 h, yellowish solid **aPTHF$_{2k}$** was obtained.

Recovered **aPTHF$_{2k}$**: yield 95%, 0.43 g; ^{1}H NMR (400 MHz, $CDCl_3$) δ 4.16 (t, *J* = 6.5 Hz, 2H), 3.45 (s, 2H), 3.41 (t, *J* = 5.3 Hz, 55H), 2.27 (s, 3H), 1.62 (dq, *J* = 6.0, 3.0 Hz, 58H).

The comparison between the ^{1}H NMR spectra of the original and recovered **aPTHF$_{2k}$** is shown in Figure 5b.

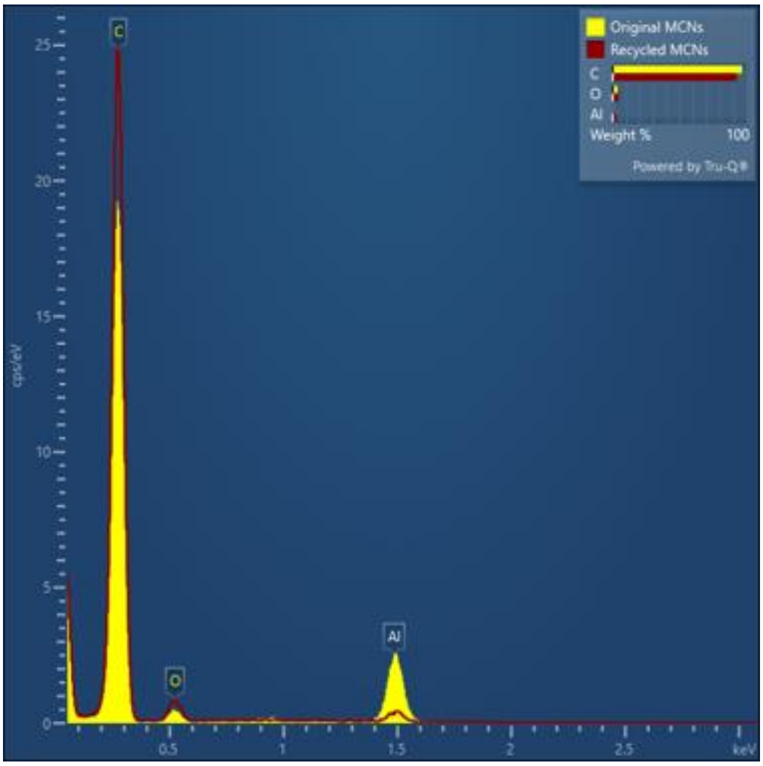

Figure S36. Overlaid EDX spectra of the original (yellow) and recycled **MCN**s (red).

Reference

[1] Z. Liu, C. Yu, C. Zhang, Z. Shi, J. Yin, *ACS Macro Lett.*, **2019**, 8, 233–238.
